# Heme as a Target for Therapeutic Interventions

**DOI:** 10.3389/fphar.2017.00146

**Published:** 2017-04-04

**Authors:** Stephan Immenschuh, Vijith Vijayan, Sabina Janciauskiene, Faikah Gueler

**Affiliations:** ^1^Institute for Transfusion Medicine, Hannover Medical SchoolHannover, Germany; ^2^Department of Pulmonology, Hannover Medical SchoolHannover, Germany; ^3^Department of Nephrology, Hannover Medical SchoolHannover, Germany

**Keywords:** heme, hemopexin, hemolysis, heme toxicity, heme oxygenases, inflammation, inflammatory diseases

## Abstract

Heme is a complex of iron and the tetrapyrrole protoporphyrin IX with essential functions in aerobic organisms. Heme is the prosthetic group of hemoproteins such as hemoglobin and myoglobin, which are crucial for reversible oxygen binding and transport. By contrast, high levels of free heme, which may occur in various pathophysiological conditions, are toxic via pro-oxidant, pro-inflammatory and cytotoxic effects. The toxicity of heme plays a major role for the pathogenesis of prototypical hemolytic disorders including sickle cell disease and malaria. Moreover, there is increasing appreciation that detrimental effects of heme may also be critically involved in diseases, which usually are not associated with hemolysis such as severe sepsis and atherosclerosis. In mammalians homeostasis of heme and its potential toxicity are primarily controlled by two physiological systems. First, the scavenger protein hemopexin (Hx) non-covalently binds extracellular free heme with high affinity and attenuates toxicity of heme in plasma. Second, heme oxygenases (HOs), in particular the inducible HO isozyme, HO-1, can provide antioxidant cytoprotection via enzymatic degradation of intracellular heme. This review summarizes current knowledge on the pathophysiological role of heme for various diseases as demonstrated in experimental animal models and in humans. The functional significance of Hx and HOs for the regulation of heme homeostasis is highlighted. Finally, the therapeutic potential of pharmacological strategies that apply Hx and HO-1 in various clinical settings is discussed.

## Introduction

**Figure 1:**
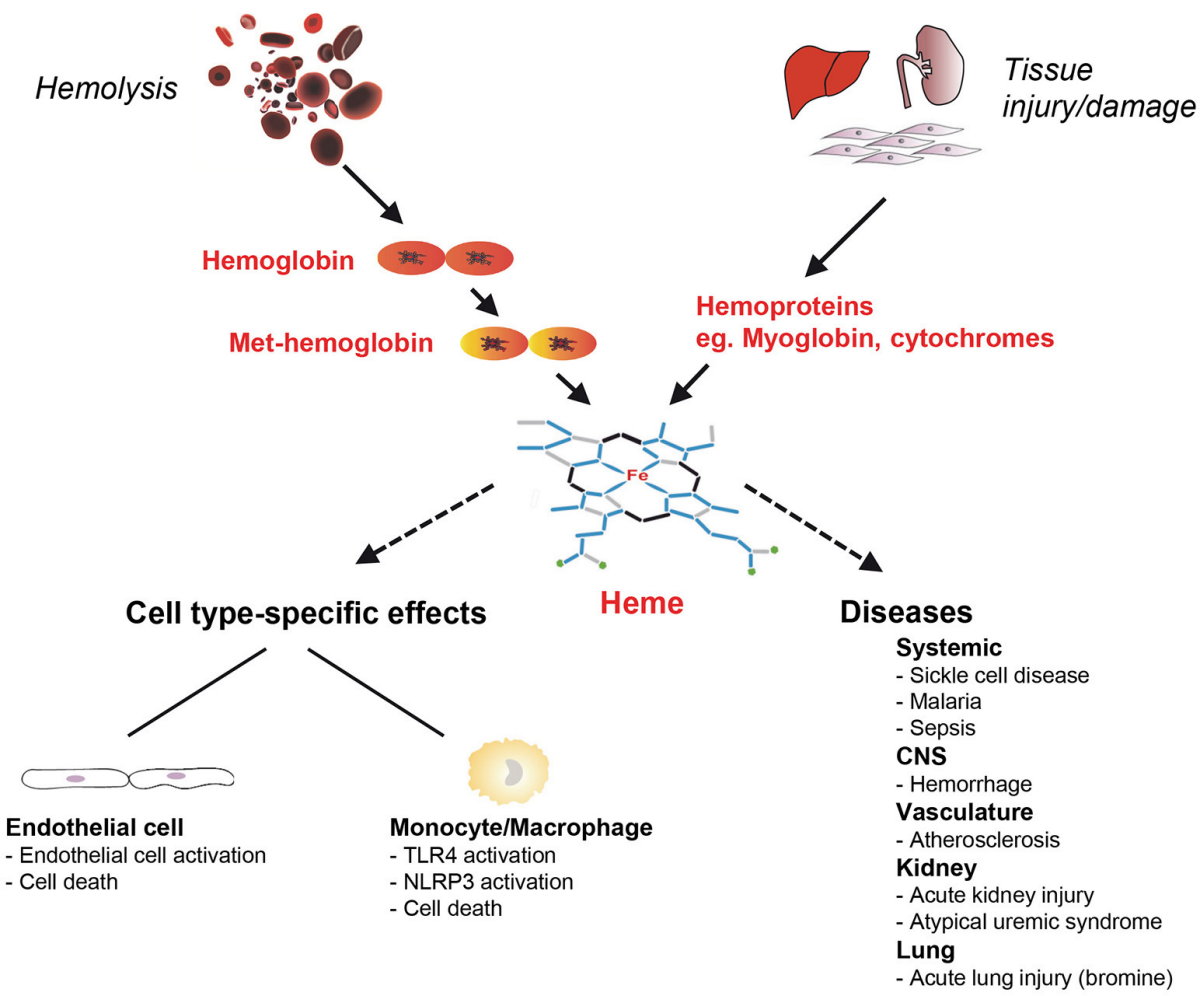
**Schematic on cell-type specific effects of heme toxicity and its role in the pathogenesis of diseases**. Free heme can arise in hemolysis from cell-free hemoglobin (Hb) oxidized to Met-Hb and in tissue damage and injury from intracellular hemoproteins that are released from cells such as myoglobin. Heme has pro-oxidant, pro-inflammatory and cytotoxic effects and can cause cell type-specific effects in endothelial cells and monocytes/macrophages. Heme is involved in the pathogenesis of various hemolytic diseases including sickle cell disease (SCD) and malaria, but also in disorders that are not typically associated with hemolysis. *CNS*, central nervous system; NLRP3, nucleotide-binding domain and leucine-rich repeat pyrin 3 containing; TLR, toll-like receptor.

Heme is a ubiquitous molecular complex of iron and the tetrapyrrole protoporphyrin IX. When bound to hemoproteins, heme plays an essential role for numerous biological processes in aerobic organisms, which range from reversible oxygen binding to electron transport of the respiratory chain (Wagener et al., 2003; Hamza and Dailey, 2012). However, despite its well-established physiological functions, heme can be harmful and critically involved in the pathogenesis of various diseases. In pathological conditions, such as hemolysis and tissue damage, large amounts of hemoglobin (Hb), myoglobin and other hemoproteins are released into the circulation (Reeder, 2010; Schaer et al., 2012). Specifically, in hemolytic disorders cell-free Hb released from damaged red blood cells (RBCs) can rapidly exhaust the binding capacity of the scavenger protein haptoglobin (Hp) that neutralizes the pro-oxidant effects of extracellular Hb (Schaer et al., 2012). Heme iron in non Hp-bound cell-free Hb is rapidly oxidized from the Fe^2+^ to the Fe^3+^ state and forms met-Hb (also termed oxy-Hb), which then releases free heme (Bunn and Jandl, 1968; Hebbel et al., 1988; Balla et al., 1993; Figure 1). After exceeding the binding capacity of the heme scavenger hemopexin (Hx), free heme accumulates in the plasma (Muller-Eberhard and Cleve, 1963; Muller Eberhard, 1970; Tolosano and Altruda, 2002). Free heme may also arise from myoglobin and other hemoproteins released from damaged cells during tissue injury (Figure 1). High concentrations of free heme can be cytotoxic via the formation of reactive oxygen species (ROS) (Kumar and Bandyopadhyay, 2005; Larsen et al., 2012; Roumenina et al., 2016). Moreover, due to the lipophilic structure heme can intercalate with cell membranes resulting in lipid and protein peroxidation or DNA damage (Aft and Mueller, 1983, 1984; Vincent, 1989).

In this review, we will summarize the current understanding of how heme toxicity is involved in the pathogenesis of various clinical conditions and diseases, which include not only classical hemolytic diseases, such as sickle cell disease (SCD) and malaria, but also non-typical hemolytic diseases, such as severe sepsis and atherosclerosis. The roles of heme neutralization via the plasma scavenger protein Hx and heme-degrading heme oxygenases (HOs) are highlighted. Finally, the therapeutic potential of Hx and that of HOs is discussed in clinically relevant conditions.

## Physiological functions of heme

The tetrapyrrole heme (iron protoporphyrin IX) not only serves key physiological functions in mammalians, but also in bacteria and plants. Heme is a prosthetic group in numerous hemoproteins, in which it occurs in its non-covalently or covalently bound form (Ponka, 1999; Wagener et al., 2003; Reeder, 2010; Hamza and Dailey, 2012). For example, heme *b*, which is the most abundant form of heme, is non-covalently bound to the hemoproteins Hb and myoglobin. Both hemoproteins are of major importance for reversible binding and transport of oxygen (Reeder, 2010). Moreover, covalently-bound heme *c* in cytochrome c is critical for electron transfer in the mitochondrial respiratory chain (Chance, 1967). Similarly, heme is a functionally important compound in multiple other hemoproteins, such as cytochrome-P450s, soluble guanylate cyclase, cyclooxygenase-2, inducible nitric oxide synthase or NADPH oxidases, all of which are key enzymes for cellular homeostasis (Mense and Zhang, 2006). In addition to its role in hemoproteins, a minor portion of intracellular heme is available as so-called “free” heme, which is considered to be loosely associated to proteins other than hemoproteins (Ponka, 1997; Chiabrando et al., 2014; Soares and Bozza, 2016) and is also known as the labile or non-determined heme pool. As proposed for hepatocytes more than four decades ago, free heme has functional regulatory relevance for cellular metabolic events (Granick et al., 1975). Although available only to a minor extent under normal conditions, free heme is an important signaling molecule for cellular sensing of gases (e.g., oxygen, carbon monoxide or nitric oxide) or regulation of the circadian rhythm (Granick et al., 1975; Mense and Zhang, 2006; Burris, 2008; Girvan and Munro, 2013).

Due to the multiple functions of heme, regulation of intra- and extracellular heme homeostasis is of major physiological significance and is tightly controlled at various levels. First of all, enzymatic synthesis and degradation of heme is mediated via a complex system that is controlled by feedback mechanisms in a cell type-specific manner (Abraham et al., 1983; Ponka, 1997; Ryter and Tyrrell, 2000; Wijayanti et al., 2004). Moreover, heme transporters, such as the heme exporters Feline leukemia virus subgroup C receptor 1a (FLVCR1a) or ATP-binding cassette subfamily G member2 (ABCG2) and the heme importer FLVCR2, mediate shuttling of heme across cellular membranes. Finally, a number of heme binding proteins (HBPs), which are discussed in more detail below, can reversibly bind and release heme to control its intra- and extracellular homeostasis (Muller Eberhard and Nikkilä, 1989; Chiabrando et al., 2014). Comprehensive overviews on the multiple physiological functions of heme have been previously given by other authors (Chance, 1967; Ponka, 1999; Wagener et al., 2003; Mense and Zhang, 2006; Hamza and Dailey, 2012; Girvan and Munro, 2013).

## Heme toxicity in various cell types

Although, heme toxicity applies to all cells and tissues, distinct cell types can be differentially affected by its harmful effects. In RBCs as the major population of heme-containing cells, heme critically affects aging via long-term intercalation and destabilization of membranes (Solar et al., 1991; Rifkind and Nagababu, 2013). Activation of neutrophils by heme leads to oxygen radical production, chemotaxis and the formation of neutrophil extracellular traps (Chen et al., 2014). Importantly, various cell types exhibit different sensitivities to the toxicity of heme. For example, heme causes cell death in cultures of endothelial cells at markedly lower concentrations as compared to macrophages or epithelial cells (Vijayan and Immenschuh, unpublished observations) suggesting that various modes of auto-protection against heme toxicity are operative in divergent types of cells. Moreover, in distinct pathophysiological settings, cells may exhibit context-specific spatial and temporal regulatory patterns of adaptation. Herein, we focus on heme toxicity in endothelial cell and monocyte/macrophage models (Figure 1).

### Endothelial cells

The endothelium, in particular the vascular endothelium, has important homeostatic functions and is involved in the pathogenesis of various diseases including inflammatory cardiovascular disorders (Pober et al., 2009). In hemolysis the vascular endothelium may encounter increased concentrations of heme, which can be as high as 100 μM (Muller-Eberhard et al., 1968; Balla et al., 1993; Wagener et al., 2003). Autoprotection of the endothelium against heme toxicity is of critical importance under hemolytic conditions, because heme can sensitize cell cultures of endothelial cells to prooxidant damage by granulocytes or toxic ROS (Balla et al., 1991). Moreover, heme induces inflammatory activation of endothelial cells *in vitro* and *in vivo* as indicated by the up-regulation of inducible adhesion molecules including vascular cell adhesion molecule (VCAM)-1 or intercellular cell adhesion molecule (ICAM)-1 (Wagener et al., 1997, 2001) as well as release of von Willebrand factor and P-selectin from Weibel-Palade bodies (Belcher et al., 2014; Figure 1). It is also important to note that auto-protection of endothelial cells against heme toxicity is markedly impaired in murine and human genetic deficiency of HO-1, which is the inducible isoform of the heme-degrading enzyme HO (discussed in more detail below) (Tenhunen et al., 1968; Abraham et al., 1988; Maines, 1997). In murine and human HO-1 deficiency the endothelium is afflicted by major pro-oxidant damage and detachment from glomerular basal membranes (Poss and Tonegawa, 1997a; Yachie et al., 1999; True et al., 2007). Remarkably, endothelial HO-1 gene expression is inversely linked with platelet endothelial cell adhesion molecule (PECAM)-1, a key endothelial surface receptor, suggesting a specific interrelation between these two proteins in the endothelium (Saragih et al., 2014).

### Macrophages/monocytes

Macrophages are key cells of the immune system controlling homeostasis of immunological regulation, host defense and wound healing (Mosser and Edwards, 2008). Macrophages are resistant to relatively high concentrations of heme in comparison to endothelial cells. A major function of spleen and liver tissue macrophages is the elimination of circulating senescent RBCs. Thus, it is not surprising that these cells exhibit constitutive high expression of HO-1 *in vivo* to protect against heme toxicity (Bissell et al., 1972; Immenschuh et al., 1999, 2003). Interestingly, differentiation of liver and spleen tissue macrophages is modulated via a heme-dependent pathway that involves the nuclear heme-regulated protein BTB domain and CNC homolog 1 (Bach1) (Haldar et al., 2014). Major relevance of macrophages for heme recycling and iron homeostasis has also been demonstrated in HO-1 knockout mice, that exhibit reduced numbers and function of erythrophagocytosing macrophages (Kovtunovych et al., 2010). It is important to note that heme, but not its analogs or precursors, activates murine macrophages via toll-like receptor (TLR)-4 (Figueiredo et al., 2007). Moreover, cell-free Hb and its derivative heme, which can arise from damaged RBCs, synergistically up-regulated TLR-dependent pro-inflammatory responses in primary mouse bone-marrow derived macrophages (Lin et al., 2010). The underlying mechanistic details on the interactions of heme with TLRs and the potential intracellular signaling cascades that may mediate these functional interactions are under investigation (Dutra and Bozza, 2014; Soares and Bozza, 2016). More recently, heme has also been shown to activate the nucleotide-binding domain and leucine-rich repeat pyrin 3 containing (NLRP3) inflammasome in murine macrophages *in vivo* and *in vitro* (Dutra et al., 2014; Figure 1).

In conclusion, various cell types are differentially affected by the toxicity of heme and play distinct roles for the control of local and systemic heme homeostasis in physiological and pathophysiological conditions.

## Role of heme in the pathogenesis of diseases

**Figure 2:**
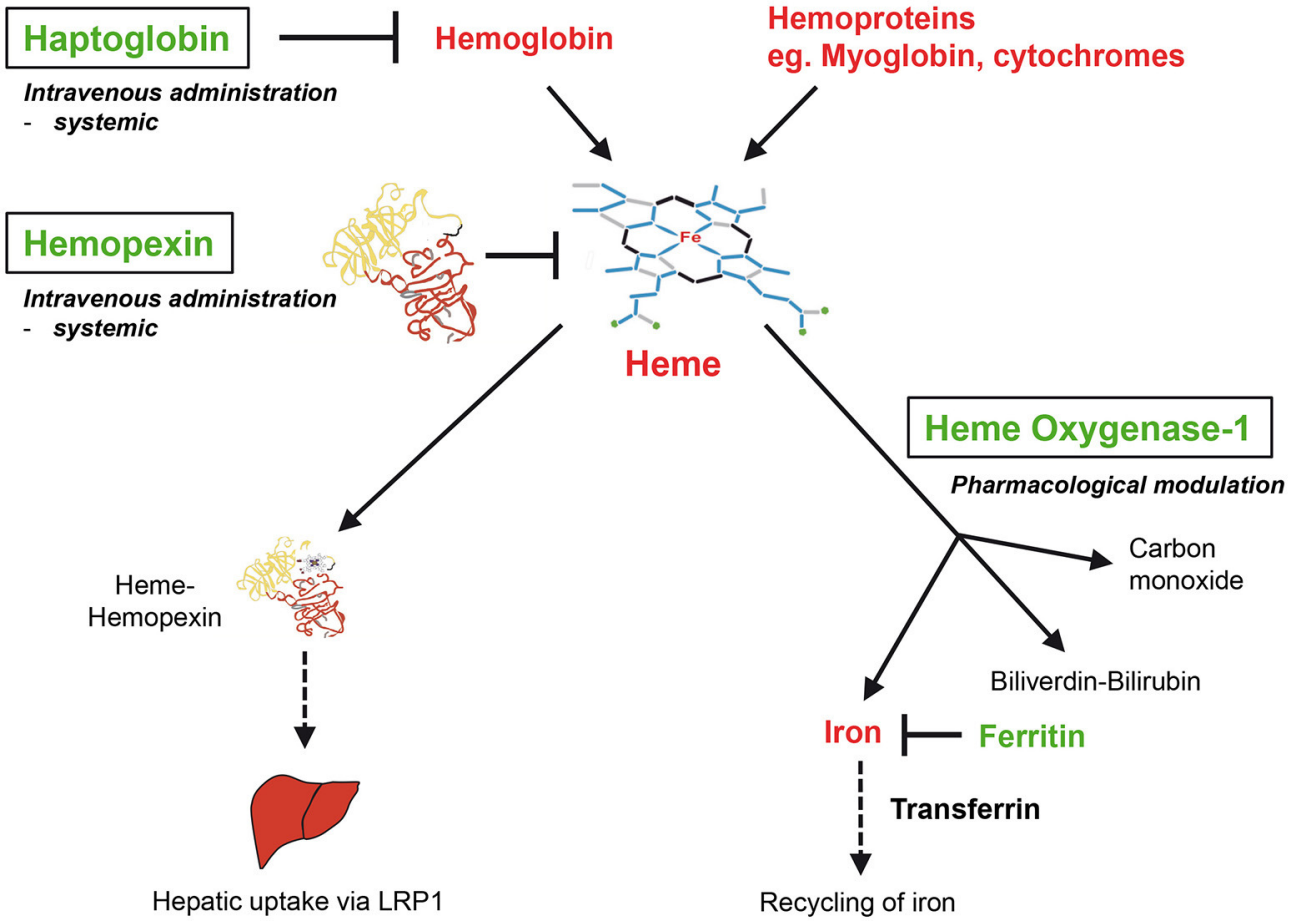
**Therapeutic interventions for the neutralization of heme**. The antioxidant scavenger proteins haptoglobin (Hp) and hemopexin (Hx) bind and neutralize extracellular Hb and free heme in plasma, respectively. HO-1 is the inducible isoform of HOs, which enzymatically degrade intracellular heme to produce iron, carbon monoxide and biliverdin, which is converted into bilirubin by biliverdin reductase. Hx and Hp may be applied as a potential heme-neutralizing therapy via systemic intravenous administration. Potential therapies of HO-1 may be performed via targeted pharmacological induction. LRP1, low density lipoprotein receptor-related protein 1.

A growing number of reports has demonstrated a critical role of heme toxicity in the pathogenesis of various diseases (Muller Eberhard and Nikkilä, 1989; Ryter and Tyrrell, 2000; Wijayanti et al., 2004; Kumar and Bandyopadhyay, 2005). In severe hemolysis or tissue injury excess amounts of extracellular Hb, myoglobin and free heme overwhelm the binding capacity of the plasma scavengers Hp and Hx and cause systemic or local heme-dependent pathologies (Figures 1, 2). Besides, heme can have indirect toxic effects in the pathogenesis of atherosclerosis (Nagy et al., 2010) or irritant gas-induced acute lung injury (Aggarwal et al., 2016). Heme may also act as a secondary hit that causes disease manifestation of a preexisting clinical risk constellation (Frimat et al., 2013). In this section, different roles of heme for the pathophysiology of experimental animal models and human disorders are discussed.

### Animal models of human diseases

#### Hemolytic diseases: SCD and malaria

Important findings on the pathogenic potential of heme have been reported in experimental mouse models of SCD and malaria. Although of different origin, pathologies in both disorders are primarily linked to large amounts of hemoproteins released from damaged RBCs. SCD is a genetic disorder caused by an amino acid exchange in the Hb β-chain, which leads to the production of abnormally shaped sickle cells prone to intravascular hemolysis (Ingram, 1957). Experimental SCD mouse models seem to exhibit typical signs of vascular inflammation (Belcher et al., 2003). Furthermore, as indicated by leukocyte infiltration and thrombosis, heme toxicity is likely associated with phenotypical alterations of the vascular endothelium (Belcher et al., 2014; Keleku-Lukwete et al., 2015). Independently, heme was responsible for pro-inflammatory M1 polarization of macrophages (Vinchi et al., 2016), formation of neutrophil extracellular traps (Chen et al., 2014), and triggering of acute chest syndrome (Ghosh et al., 2013) in SCD mice. In malaria, which is a protozoan disease, hemolysis is caused by infection with and replication of *Plasmodium* in RBCs (Miller et al., 2002). In mouse models of experimental malaria release of extracellular Hb and heme from damaged RBCs also had major pro-oxidant and pro-inflammatory effects. Hemolysis-derived heme was directly involved in inflammatory pathologies of experimental murine cerebral and non-cerebral malaria (Pamplona et al., 2007; Seixas et al., 2009).

#### Central nervous system (CNS) hemorrhage

CNS hemorrhage occurring spontaneously, like in subarachnoid hemorrhage and stroke or because of traumatic injuries, can be closely associated with the toxicity of cell-free Hb and heme. In a feline model exposure to cell-free Hb caused marked pro-oxidant damage of nerve cells *in vivo*, which was primarily due to lipid peroxidation (Sadrzadeh et al., 1987). Similar findings have been reported in an experimental rabbit model of preterm pup intraventricular hemorrhage, in which Hb and heme led to inflammatory changes and cell death in affected tissues (Gram et al., 2014). Furthermore, heme toxicity played an important role in the pathogenesis of brain injury in a mouse model of intracerebral injection of cell-free Hb (Ma et al., 2016).

#### Sepsis

Sepsis and its more complicated manifestations, severe sepsis and septic shock, are characterized by an excessive systemic inflammatory response to various acute injuries (Bone, 1991; Gotts and Matthay, 2016). The pathophysiology of this complex disorder is not understood in detail (Angus and van der Poll, 2013). Recently, cell-free Hb and heme have been shown to be involved in the pathogenesis of severe sepsis in a mouse model of cecal ligation and puncture polymicrobial sepsis *in vivo* (Larsen et al., 2010). Similarly, pro-inflammatory effects of extracellular Hb and heme from degraded erythrocytes worsened the survival rate in an experimental rat model of *E. coli-*mediated sepsis (Griffiths et al., 1995).

#### Transfusion of RBCs

Numerous reports have associated transfusions of packed RBCs after prolonged storage with increased morbidity and mortality in trauma-induced hemorrhage among other conditions. Detrimental effects of stored RBCs have been associated with the so-called “storage lesion,” which is characterized by RBC alterations including loss of metabolites, decreased cell volume with accompanying formation of echinocytes and release of free Hb due to hemolysis (Lelubre et al., 2009). The storage lesion has been directly linked to the toxicity of cell-free Hb and heme in a model of guinea pigs, in which transfusion of senescent RBCs was more harmful if compared with fresh RBC preparations (Baek et al., 2012). Similarly, others have demonstrated that heme toxicity due to RBC storage lesion markedly aggravated the outcome in two independent mouse models of trauma-induced hemorrhage (Stapley et al., 2015; Graw et al., 2016). Finally, transfusion of senescent RBCs aggravated inflammation and worsened outcome in a canine model of infectious pneumonia (Wang et al., 2012a).

#### Disorders in kidney, heart, and lung

Heme toxicity is also involved in the pathogenesis of diseases in solid organs such as kidney. For example, experimental rhabdomyolysis, in which large amounts of intracellular hemoproteins such as myoglobin are released, cause heme-dependent acute kidney injury (AKI) (Nath et al., 1992). Moreover, the detrimental pro-oxidant effects of hemoproteins were shown to be directly involved in kidney cell damage *in vitro* and *in vivo* (Nath et al., 1995). More recent studies in mouse models have extended these earlier findings by demonstrating that the heme degradation product iron and the iron-sequestering protein ferritin are critically associated with heme-dependent renal injury (Zarjou et al., 2013; Bolisetty et al., 2015). Comprehensive overviews on the pathophysiology of heme toxicity in kidney diseases have been previously given (Tracz et al., 2007; Lever et al., 2016). As to cardiac disorders increased levels of heme seem to aggravate ischemia-reperfusion injury in experimental murine heart disease. This novel finding suggests an important role for heme in the pathogenesis of ischemic cardiomyopathy (Sawicki et al., 2015). Remarkably, heme toxicity was also critically involved in acute lung injury due to bromine inhalation in a mouse model (Aggarwal et al., 2016).

### Human diseases

Mouse models are useful to explore basic pathophysiological disease mechanisms and to investigate novel therapeutic interventions. However, species-specific differences in fundamental regulatory systems between mouse and human have been pointed out, e.g., for the immune system (Mestas and Hughes, 2004), indicating that experimental findings in animal models may not always be translatable into clinical applications. It is also important to note that human disorders are in general more complex in comparison to mouse disease models, which are frequently caused by single mechanisms (Warren et al., 2015). Major discrepancies in various inflammatory conditions of mouse and human have been reported for genomic responses and are controversially discussed (Seok et al., 2013).

#### SCD and malaria

Patients with hemolytic disorders such as SCD exhibit increased serum levels of heme (Muller-Eberhard et al., 1968) and develop acute and/or chronic manifestations of heme toxicity (Nath and Katusic, 2012). Remarkably, in SCD patients heme-carrying RBC membrane microparticles were responsible for endothelial cell damage (Camus et al., 2015) and heme specifically affected T cell polarization via interaction with CD16+ monocytes (Zhong et al., 2014). A direct pathogenic role for heme toxicity has also been demonstrated in human malaria. Heme markedly down-regulated prostaglandin and transforming growth factor-β production in malaria (Andrade et al., 2010). Moreover, increased concentrations of heme were associated with higher susceptibility to malaria (Mendonca et al., 2012) and disease outcome was linked with systemic levels of extracellular heme in these patients (Elphinstone et al., 2016).

#### Sepsis

Similar to findings in mouse models, heme appears to play a role in the pathogenesis of severe sepsis in humans as indicated by lower serum levels of Hx, which decrease due to increased levels of heme (Larsen et al., 2010). Accordingly, poor outcome in sepsis patients has been associated with decreased serum levels of Hp and Hx (Janz et al., 2013; Lin et al., 2015). In the context of sepsis it is important to note that mice exhibit markedly higher tolerance to endotoxin relative to humans (Schaedler and Dubos, 1961).

#### Transfusion of packed RBCs

Transfusion of packed RBCs after prolonged storage has been associated with an increased risk of death in critically ill patients (Wang et al., 2012b). Therefore, the potential risk of transfusing senescent RBCs needs to be addressed by prospective controlled studies to clarify this issue in more detail.

#### Cardiac diseases (atherosclerosis, ischemic cardiomyopathy, and heart failure)

Hemolysis-derived heme may be indirectly involved in the pathogenesis of atherosclerosis. The oxidation of lipoproteins and other plasma proteins by pro-oxidant iron from cell-free Hb and heme plays an important pathophysiological role for the complex sequence of vascular events that cause atherosclerosis (Jeney et al., 2002, 2014; Nagy et al., 2010). In clinical studies on chronic ischemic cardiomyopathy increased levels of heme have been observed in cardiac biopsies from patients with failing hearts (Sawicki et al., 2015). Independently, up-regulation of heme levels have been associated with a worse clinical outcome in patients with heart failure (Khechaduri et al., 2013). Interestingly, heme toxicity has recently been implicated in the pathogenesis of heart failure via affecting the contractile function of cardiomyocytes (Alvarado et al., 2015).

#### Atypical hemolytic uremic syndrome

A recent clinical study of patients with atypical hemolytic uremic syndrome (a rare thrombotic microangiopathy primarily observed in the kidney) identified heme as a critical secondary hit that triggers the clinical manifestation of this disorder. Specifically, heme in plasma from patients with atypical hemolytic uremic syndrome activated the alternative pathway of the complement cascade, which in turn caused endothelial cell activation (Frimat et al., 2013). These findings suggest that heme may have similar effects in the pathophysiology of other vascular inflammatory diseases.

### Diagnostic tests for determining free heme levels in plasma and tissues

A major hurdle for a better understanding of heme toxicity in clinical practice is the lack of a reliable diagnostic test for determining levels of free heme in plasma and tissue biopsies. Currently, the severity of hemolysis and the potential toxicity of heme can only be indirectly estimated via determining plasma concentrations of Hp and Hx, both of which inversely correlate with increased levels of cell-free Hb and free heme in severe hemolysis (Muller-Eberhard et al., 1968). Thus, a diagnostic test for determining heme concentrations in biological fluids and tissues is urgently needed.

In conclusion, experimental animal models of human disease and studies in human disorders confirm the pertinent pathophysiological role of heme toxicity; however, the appropriate test systems for detection of free heme are still missing.

## Protection against heme toxicity via hemopexin and the heme oxygenase system - its therapeutic potential

### Regulation of physiological heme homeostasis by hemopexin (Hx) and heme oxygenases (HOs)

In mammalians heme homeostasis is primarily controlled by two regulatory systems. Firstly, the plasma scavenger protein Hx neutralizes and eliminates excess free heme from the circulation. Secondly, intracellular heme is primarily enzymatically degraded via the heme-catabolizing HOs, in particular by its inducible isoform HO-1.

#### Hemopexin (Hx)

The plasma protein Hx binds non-covalently heme with the highest affinity of any known protein (K_D_ 10^−14^) (Muller-Eberhard and Cleve, 1963; Muller Eberhard, 1970; Tolosano and Altruda, 2002) via its characteristic heme-binding pocket (Paoli et al., 1999). The major function of Hx appears to be neutralization and scavenging of excess free heme from the circulation. Up-take of heme-Hx complexes in the liver (Potter et al., 1993) is mediated via the scavenger receptor low-density lipoprotein receptor-related protein-1 (LRP1, synonymous with CD91) (Hvidberg et al., 2005; Vercellotti et al., 2016). Hx belongs to the acute-phase reactants, which include a number of plasma proteins such as C-reactive protein, α2-macroglobulin and α1-antitrypsin that are up-regulated in the liver as part of a systemic inflammatory response (Heinrich et al., 1990; Baumann and Gauldie, 1994). Hx is induced during the acute-phase response in rodents, but not in human, which might be due to evolutionary differences in rodent and human Hx gene promoters (Heinrich et al., 1990; Poli et al., 1990; Immenschuh et al., 1994). This species-specific difference of Hx gene regulation in rodents and humans correlates with the recently reported findings that Hx is up-regulated during sepsis in mice, but down-regulated in humans (Lin et al., 2015). Hx knockout mice exhibit a normal phenotype in non-challenged conditions, but are afflicted with heme-mediated renal and hepatic damage in conditions of experimental hemolysis (Tolosano et al., 1999; Vinchi et al., 2008). Remarkably, heme-mediated pathologies are aggravated in Hx/Hp double knockout mice (Tolosano et al., 2002) suggesting that these two plasma proteins represent a sequential protection system against the detrimental effects of hemolysis (Deuel et al., 2015; Smith and McCulloh, 2015; Figure 2).

#### Other heme binding proteins (HBPs)

In addition to Hx, other HBPs are likely to be involved in the regulation of systemic extracellular and also intracellular homeostasis of heme and may counteract its pro-oxidant effects. The functional significance of most known HBPs for neutralization and transport of heme is only incompletely understood. However, it has been pointed out that the specific heme-protein interactions of a given HBP determine its protective potential against the pro-oxidant effects of heme, respectively (Vincent et al., 1988; Vincent, 1989). Another extracellular HBP with major physiological significance is albumin, which binds heme with markedly lower affinity than Hx (K_D_ 1.2 × 10^−8^) (Little and Neilands, 1960), but exhibits markedly higher plasma concentrations relative to Hx (Adams and Berman, 1980) (Table 1). Other known extracellular plasma HBPs are α1-microglobulin (Allhorn et al., 2002) and α1-antitrypsin (Karnaukhova et al., 2012) (Table 1). Intracellular binding of heme by HBPs may not only protect against its potential pro-oxidant toxicity, but may be also involved in trafficking of heme between different cell compartments (Muller Eberhard and Nikkilä, 1989; Liem et al., 1994; Yuan et al., 2016). Thus, the specific functional roles of intracellular candidate HBPs in mammalians such as glutathione-S-transferases, heme-binding protein/ liver fatty-acid binding protein, heme-binding protein 23/peroxiredoxin 1, p22 heme binding protein and glyceraldehyde-3-phosphate dehydrogenase (Harvey and Beutler, 1982; Vincent and Muller Eberhard, 1985; Iwahara et al., 1995; Taketani et al., 1998; Chakravarti et al., 2010) need to be investigated in more detail (Table 1).

**Table 1:** **Heme binding proteins (HBPs) in mammalians**.

**Heme binding protein**	**Concentration**	**K_D_**	**References**
**EXTRACELLULAR**			
Hemopexin	0.6–1.2 g/L	1 × 10^−14^	Muller-Eberhard and Cleve, 1963; Muller Eberhard, 1970
Albumin (human)	35–53 g/L	1.2 × 10^−8^	Little and Neilands, 1960; Adams and Berman, 1980
α1-Microglobulin	0.03 g/L	1 × 10^−6^	Allhorn et al., 2002
α1-Antitrypsin	1.3–2.5 g/L	2 × 10^−8^	Karnaukhova et al., 2012
**INTRACELLULAR**			
Glutathione-S transferases	3–5% of total protein (liver)	1 × 10^−7^	Harvey and Beutler, 1982
Heme binding protein/Liver fatty acid binding protein	3–5% of total protein (liver)	2 × 10^−7^	Vincent and Muller Eberhard, 1985
Heme binding protein 23/peroxiredoxin 1	0.1% of total protein (liver)	5.5 × 10^−8^	Iwahara et al., 1995
p22 heme-binding protein	n. d.	2.5 × 10^−8^	Taketani et al., 1998
Glyceraldehyde-3-phosphate dehydrogenase	10% of total protein (skeletal muscle)	n. d.	Chakravarti et al., 2010

#### Heme oxygenases (HOs)

Enzymatic degradation of intracellular heme is primarily mediated via the HO system independent of P450s (Tenhunen et al., 1968; Maines and Kappas, 1974; Maines, 1997). The HO reaction has three major products: the signaling gas carbon monoxide (CO), iron and biliverdin, which is subsequently converted into bilirubin by biliverdin reductase (Kutty and Maines, 1981; Figure 2). Two genetically distinct isoforms of HO are known. HO-2 represents the constitutive non-inducible isoform and is primarily expressed in brain and testes (Trakshel et al., 1986). By contrast, the inducible HO isoform, HO-1, is expressed in almost all cells and tissues, and is highly up-regulated by heme or other stress stimuli to provide protection against oxidative damage and apoptosis (Maines, 1997; Ryter et al., 2006). The cytoprotective functions of HO-1 are directly linked with that of the iron-sequestering protein ferritin, which is co-ordinately up-regulated with HO-1 and neutralizes the pro-oxidant effects of the HO product iron (Balla et al., 1992; Figure 2). Importantly, genetic deficiency in mouse models and/or genetic deficiency and functional defects of HO-1 in humans are associated with major pro-oxidant and pro-inflammatory pathologies and with disturbed iron metabolism (Poss and Tonegawa, 1997a,b; Yachie et al., 1999; Kapturczak et al., 2004; Greil et al., 2016). In contrast, deficiency of the HO-2 gene in mice does not cause major heme-dependent pathologies (Poss et al., 1995) suggesting that HO-1 might be the more critical HO isozyme for counter-acting heme toxicity (Wagener et al., 2003; Kumar and Bandyopadhyay, 2005; Gozzelino et al., 2010). Studies in a conditional HO-1 knockout mouse model with targeted deletion in myeloid cells revealed a critical role of HO-1 for the regulation of innate immunity (Tzima et al., 2009). It is also interesting to point out that patterns of mouse and human HO-1 gene expression are differentially regulated in a species-specific manner (Sikorski et al., 2004). For example, HO-1 gene expression is up-regulated by lipopolysaccharide in mouse macrophages, but down-regulated in human macrophages (Miyazaki et al., 2010; Dorresteijn et al., 2015). Notably, the proximal promoter region of the human HO-1 gene, but not that of the mouse, contains a GT-microsatellite polymorphism, which may be responsible for interspecies-specific regulatory differences (Yamada et al., 2000). Finally, higher inducibility of the human HO-1 gene by oxidative stress has been associated with protection against cardiovascular disorders (Exner et al., 2004; Pechlaner et al., 2015) and against acute chest syndrome in SCD (Bean et al., 2012). Comprehensive overviews on the multiple roles of the HO system in health and disease have previously been given (Maines, 1997; Immenschuh and Ramadori, 2000; Abraham and Kappas, 2005; Ryter et al., 2006).

### Applications of hemopexin and heme oxygenases for potential therapeutic interventions

Various therapeutic strategies that may apply specific neutralization of heme toxicity via either Hx or HOs are conceivable in clinical settings and will be discussed in the following.

#### Hemopexin

Hx protects against heme toxicity not only in animal models of hemolytic disorders such as SCD and malaria (Ghosh et al., 2013; Belcher et al., 2014), but also in other diseases, which are not typically associated with hemolysis such as sepsis (Larsen et al., 2010), cardiac disease (Vinchi et al., 2013) and bromine-induced acute lung injury (Aggarwal et al., 2016) (Table 2). It is plausible that the salutary effects of Hx described in animal disease models are translatable into the clinic. Hx might also have a prophylactic potential in clinical risk constellations, in which heme triggers overt disease manifestation. For example, pretreatment with Hx before transfusion of senescent RBCs improved the outcome in different mouse models of trauma-induced hemorrhage via neutralization of heme (Stapley et al., 2015; Graw et al., 2016). The role of Hx administration as a preventive intervention is also supported by findings of others (Tolosano et al., 2010). Hence, it is conceivable that patients that might benefit from prophylactic Hx treatment are those with comorbidities (i.e., diabetes mellitus, hypertension and older age) and an increased risk for AKI, with a high likelihood for the need of RBC transfusion during major surgery. Notably, in Japan Hp has been approved for medical indications, in which renal protection is required such as in massive transfusion or thermal injury (Schaer et al., 2012). Due to the known protective effects of Hx and Hp in hemolysis it is reasonable to assume that combined treatment with these two proteins may provide a synergistic protection in clinical settings of severe hemolysis (Schaer et al., 2014; Deuel et al., 2015; Graw et al., 2016).

**Table 2:** **Hemopexin (Hx) as a therapy against heme toxicity in experimental disease models**.

**Experimental disease model**	**Therapeutic strategy**	**Protective effect**	**References**
Heme-induced acute chest syndrome in SCD mouse (C57BL/6)	Injection of single dose of recombinant human Hx (1 mg/mouse)	Heme clearance from plasma Prevention of acute lung injury Reduced mortality Hx treatment at the time of haemolytic crisis onset prevents respiratory failure	Ghosh et al., 2013
Cecal-ligation puncture induced severe sepsis in mouse (BALB/c)	Injection of multiple doses of rabbit Hx (50 mg/kg)	Reduced tissue damage. Reduced mortality	Larsen et al., 2010
SCD mouse (C57BL/6)/β-thalassemia mouse (C57BL/6)	Injection of multiple doses of Hx (0.7 mg/mouse)	Attenuation of endothelial cell activation Decreased iron accumulation in the heart Normalized blood pressure and improved cardiac function	Vinchi et al., 2013
SCD mouse (NY1DD (C57BL/6))	Liver-targeted mouse Hx gene delivery by *Sleeping Beauty* transposase system	Increased expression of Nrf2 and HO-1 Reduced heme induced microvascular stasis	Vercellotti et al., 2016
Resuscitation after hemorrhagic shock in mouse (C57BL/6)	Injection of single dose of Hx (7.5 mg/mouse)	Reduced circulating free heme levels Reduced expression of pro-inflammatory cytokine IL-6 Reduced mortality	Graw et al., 2016
Resuscitation after trauma-induced hemorrhage in mouse (C57BL/6)	Injection of single dose of Hx (0.5 mg/mouse)	Decreased BAL protein levels Reduced mortality	Stapley et al., 2015

*BAL, bronchoalveolar lavage; SCD, sickle cell disease*.

##### Pharmacological applications of Hx

Intravenous administration of Hx appears to be a straightforward approach to neutralize free heme toxicity in hemolytic conditions. Thus, Hx may be applied as a human blood-derived product, similar to other plasma proteins, such as albumin, α1-antitrypsin or immunoglobulins, which are well-established therapies. Alternatively, it is also feasible that Hx might become available as a recombinant protein (Satoh et al., 1994; Hada et al., 2014). Potential side effects of Hx treatment may be caused by its known protease activity, which has been associated with inhibition of leukocyte chemotaxis and increased mortality in a mouse model (Cheung et al., 1999; Bakker et al., 2005; Spiller et al., 2011). A more recent study, however, indicated that the protease activity of Hx might not be of major clinical relevance (Lin et al., 2016).

In conclusion, the plasma protein Hx has major therapeutic potential for the neutralization of heme toxicity in various clinically relevant conditions.

#### Heme oxygenase-1

HO-1 has been shown to provide specific protection against heme toxicity in different animal models including mouse models of SCD (Belcher et al., 2006, 2010), malaria (Pamplona et al., 2007; Seixas et al., 2009) and rhabdomyolysis (Wei et al., 2011). In particular, the beneficial effects of HO-1 have been demonstrated in mouse models, in which HO-1 has been either genetically deleted or overexpressed (Table 3). However, when HO-1 is targeted for therapeutic purposes, a major challenge is that HO-1 appears to be only protective when up-regulated before onset of an experimental injury. The latter has been confirmed in rodent models of experimental pancreatitis and colitis suggesting that HO-1 might be a primary option for prophylactic interventions (Nakamichi et al., 2005; Paul et al., 2005). Comprehensive overviews on challenges with potential translational applications of HO-1 in the clinic have recently been given for renal diseases (Lever et al., 2016).

**Table 3:** **Heme oxygenase (HO)-1 as a therapy against heme toxicity in experimental disease models**.

**Experimental disease model**	**Therapeutic strategy**	**Specific protective effect**	**References**
Glycerol-induced acute kidney injury in rat (Sprague Dawley)	Preconditioning of HO-1 using hemoglobin (30 mg/100 g body weight) 20 h prior to injection with glycerol	Protection from kidney failure Reduced mortality	Nath et al., 1992
Glycerol-induced acute kidney injury in mouse (C57BL/6)	Preconditioning of HO-1 using GM-CSF (200 mg/kg body weight) for 5 consecutive days prior to injection with glycerol	Reduced blood urea nitrogen levels Reduced tissue damage Reduced mortality	Wei et al., 2011
Exposure to bromine gas in mouse (C57BL/6)	Genetic overexpression of human HO-1 using (BAC)	Attenuated bromine-induced heme levels in plasma and lung Reduced bromine-induced cytokine/chemokine levels Reduced mortality	Nagy et al., 2010
Malaria PCC-infected mouse (DBA/2)	Liver-specific overexpression of HO-1 using recombinant adenovirus	Blocked hepatic failure indicated by the decrease in AST and reduced tissue necrosis Prevented mortality	Seixas et al., 2009
S+S-Antilles SCD mouse (C57BL/6)	Liver targeted rat HO-1 gene delivery by *Sleeping Beauty* transposase system	Reduced hypoxia-induced stasis in dorsal skin fold chambers	Belcher et al., 2006

*AST, aspartate amino transferase; BAC, bacterial artificial chromosome*.

##### Pharmacological approaches that target HO-1

In contrast to the straightforward therapeutic application of Hx as systemically administered intravenous drug, potential interventions with HO-1 appear to be more complex. Multiple *in vitro* and *in vivo* studies have revealed that specific up-regulation of HO-1 via heme or low doses of Hb provide beneficial effects in various preclinical models of experimental pathological conditions such as endotoxin-mediated lung injury in rats (Otterbein et al., 1995), human immunodeficiency syndrome (Levere et al., 1991; Devadas and Dhawan, 2006) or diabetes (Ndisang et al., 2009) (for reviews see Ryter et al., 2006; Abraham and Kappas, 2008). Although, heme has been established in the clinic for treatment of acute attacks in hepatic porphyrias (Bonkowsky et al., 1971), it is important to note that administration of heme for other indications in clinical practice may be critical for two reasons. First, due to its detrimental effects, which have been discussed above, heme may aggravate inflammatory disorders. Second, heme preparations can be unstable and cause considerable side effects such as coagulopathies and vasculitis (Glueck et al., 1983; Goetsch and Bissell, 1986; Simionatto et al., 1988). Hence, the feasibility of heme as a therapy for broad medical indications appears to be questionable and needs further evaluation in clinical practice. A compound with better potential for translation into the clinic might be heme arginate, which appears to be less toxic than heme (Jeney et al., 2002) and is an approved therapy for treatment of hepatic porphyrias in various European countries (Mustajoki et al., 1986; Kordac et al., 1989). Notably, heme arginate was found to up-regulate HO-1 not only in healthy individuals (Doberer et al., 2010), but also in patients receiving deceased donor renal transplants in a recent phase IIB trial (Thomas et al., 2016). Nevertheless, further studies are required to establish the feasibility of heme arginate for clinical applications.

Further potential therapeutic applications of HO-1 may involve its cell type-specific modulation via pharmacological interventions. To this end we and others have identified regulatory pathways of HO-1 induction such as the protein kinase A and G signaling cascades in hepatocytes or the phosphatidyl-inositol-3 kinase (PI3K)/Akt cascade in mononuclear cells as putative drug targets (Immenschuh et al., 1998a,b; Wijayanti et al., 2005; Paine et al., 2010; Motterlini and Foresti, 2014). Independently, HO-1 may be regulated via established pharmaceutical compounds that have already been approved for clinical indications. For example, approved pharmacological compounds that are known to up-regulate HO-1 are statins (Grosser et al., 2004; Lee et al., 2004) and 5-aminosalicylic acid (Horvath et al., 2008). Novel candidates for targeted HO-1 up-regulation may be identified among members of the rapidly growing number of HO-1 inducing dietary and phytochemical compounds such as curcumin, quercetin or carnosol (Balogun et al., 2003; Martin et al., 2004; Peterson et al., 2009; Shen et al., 2013; Son et al., 2013) (for reviews see Ryter et al., 2006; Li et al., 2007; Abraham et al., 2009; Paine et al., 2010; Lundvig et al., 2012; Calay and Mason, 2014; Motterlini and Foresti, 2014). Finally, it is remarkable that pharmacological up-regulation of nuclear factor E2 related-factor 2 (Nrf2), which is a key nuclear regulator of HO-1, provides specific protection against heme toxicity in mouse models of SCD (Keleku-Lukwete et al., 2015; Belcher et al., 2016).

The therapeutic potential of genetic strategies that apply targeted overexpression of HO-1 for the clinic is currently not clear. Specific overexpression of HO-1 via vector-based genetic approaches in endothelial cells and adipocytes have been demonstrated to have beneficial effects in transplantation and hypertension (Chauveau et al., 2002; Cao et al., 2011, 2012; Petersen et al., 2011).

In conclusion, further studies are required before therapeutic strategies that specifically target HO-1 may become applicable in clinical practice.

## Conclusions and outlook

### Conclusions

1. Excess free heme is toxic via pro-oxidant, cytotoxic and pro-inflammatory effects.
2. Heme toxicity plays a major pathophysiological role in classical hemolytic disorders such as SCD and malaria, but also in diseases, which are not typically associated with hemolysis including sepsis and atherosclerosis.
3. The scavenger protein Hx and the HO enzyme system specifically control homeostasis and toxicity of heme in physiological and pathophysiological conditions.

### Outlook

1. Pharmacological applications of Hx and HOs are promising therapeutic options to target the toxicity of heme in various clinical settings.
2. Hx might be a near-future therapy to counteract the detrimental effects of excess extracellular free heme.
3. Potential therapeutic strategies that apply targeted modulation of HO-1 require further detailed studies.
4. A better understanding of the mechanisms that mediate heme toxicity in pathophysiology of various diseases is required to afford the development of innovative therapeutic interventions.
5. Diagnostic tests to determine free heme concentrations in plasma and tissues are necessary to achieve these goals.

## Author contributions

SI, VV, SJ, and FG participated in conceptual work and writing of the manuscript.

## Funding

Work in SI's laboratory is supported by grant IM 20/4-1 from the Deutsche Forschungsgemeinschaft, Bonn (Germany) and grant EKFS 2012_A309 from the Else Kröner Fresenius Stiftung, Bad Homburg (Germany).

### Conflict of interest statement

The authors declare that the research was conducted in the absence of any commercial or financial relationships that could be construed as a potential conflict of interest.

## References

[B1] Abraham N. G. Cao J. Sacerdoti D. Li X. Drummond G. (2009). Heme oxygenase: the key to renal function regulation. Am. J. Physiol. Renal Physiol. 297, F1137–F1152. 10.1152/ajprenal.90449.2008 19570878 PMC2781329

[B2] Abraham N. G. Friedland M. L. Lever R. (1983). Heme metabolism in hepatic and erythroid cells, in Progress in Hematology, ed Brown E. (New York: Grune and Stratton), 75–130. 6366915

[B3] Abraham N. G. Kappas A. (2005). Heme oxygenase and the cardiovascular-renal system. Free Radic. Biol. Med. 39, 1–25. 10.1016/j.freeradbiomed.2005.03.010 15925276

[B4] Abraham N. G. Kappas A. (2008). Pharmacological and clinical aspects of heme oxygenase. Pharmacol. Rev. 60, 79–127. 10.1124/pr.107.07104 18323402

[B5] Abraham N. G. Lin J. H. Schwartzman M. L. Levere R. D. Shibahara S. (1988). The physiological significance of heme oxygenase. Int. J. Biochem. 20, 543–558. 10.1016/0020-711X(88)90093-6 3292310

[B6] Adams P. A. Berman M. C. (1980). Kinetics and mechanism of the interaction between human serum albumin and monomeric haemin. Biochem. J. 191, 95–102. 10.1042/bj1910095 7470101 PMC1162185

[B7] Aft R. L. Mueller G. C. (1984). Hemin-mediated oxidative degradation of proteins. J. Biol. Chem. 259, 301–305. 6323403

[B8] Aft R. L. Mueller G. C. (1983). Hemin-mediated DNA strand scission. J. Biol. Chem. 258, 12069–12072. 6619154

[B9] Aggarwal S. Lam A. Bolisetty S. Carlisle M. A. Traylor A. Agarwal A.. (2016). Heme attenuation ameliorates irritant gas inhalation-induced acute lung injury. Antioxid. Redox Signal. 24, 99–112. 10.1089/ars.2015.6347 26376667 PMC4742996

[B10] Allhorn M. Berggard T. Nordberg J. Olsson M. L. Akerstrom B. (2002). Processing of the lipocalin α_1_-microglobulin by hemoglobin induces heme-binding and heme-degradation properties. Blood 99, 1894–1901. 10.1182/blood.V99.6.1894 11877257

[B11] Alvarado G. Jeney V. Toth A. Csosz E. Kallo G. Huynh A. T.. (2015). Heme-induced contractile dysfunction in human cardiomyocytes caused by oxidant damage to thick filament proteins. Free Radic. Biol. Med. 89, 248–262. 10.1016/j.freeradbiomed.2015.07.158 26409224

[B12] Andrade B. B. Araujo-Santos T. Luz N. F. Khouri R. Bozza M. T. Camargo L. M.. (2010). Heme impairs prostaglandin E2 and TGF-β production by human mononuclear cells via Cu/Zn superoxide dismutase: insight into the pathogenesis of severe malaria. J. Immunol. 185, 1196–1204. 10.4049/jimmunol.0904179 20562262

[B13] Angus D. C. van der Poll T. (2013). Severe sepsis and septic shock. N. Engl. J. Med. 369, 840–851. 10.1056/NEJMra1208623 23984731

[B14] Baek J. H. D'Agnillo F. Vallelian F. Pereira C. P. Williams M. C. Jia Y.. (2012). Hemoglobin-driven pathophysiology is an *in vivo* consequence of the red blood cell storage lesion that can be attenuated in guinea pigs by haptoglobin therapy. J. Clin. Invest. 122, 1444–1458. 10.1172/JCI59770 22446185 PMC3314461

[B15] Bakker W. W. Borghuis T. Harmsen M. C. A. Van den Berg A. Kema I. P. Niezen K. E.. (2005). Protease activity of plasma hemopexin. Kidney Int. 68, 603–610. 10.1111/j.1523-1755.2005.00438.x 16014037

[B16] Balla G. Jacob H. S. Balla J. Rosenberg M. Nath K. Apple F.. (1992). Ferritin: a cytoprotective antioxidant strategem of endothelium. J. Biol. Chem. 267, 18148–18153. 1517245

[B17] Balla G. Vercellotti G. M. Muller-Eberhard U. Eaton J. Jacob H. S. (1991). Exposure of endothelial cells to free heme potentiates damage mediated by granulocytes and toxic oxygen species. Lab. Invest. 64, 648–655. 2030579

[B18] Balla J. Jacob H. S. Balla G. Nath K. Eaton J. W. Vercellotti G. (1993). Endothelial-cell heme uptake from heme proteins: induction of sensitization and desensitization to oxidant damage. Proc. Natl. Acad. Sci. U.S.A. 90, 9285–9289. 10.1073/pnas.90.20.9285 8415693 PMC47552

[B19] Balogun E. Hoque M. Gong P. Killeen E. Green C. J. Foresti R.. (2003). Curcumin activates the haem oxygenase-1 gene via regulation of Nrf2 and the antioxidant-responsive element. Biochem. J. 371, 887–895. 10.1042/bj20021619 12570874 PMC1223348

[B20] Baumann H. Gauldie J. (1994). The acute phase response. Immunol. Today 15, 74–80. 10.1016/0167-5699(94)90137-6 7512342

[B21] Bean C. J. Boulet S. L. Ellingsen D. Pyle M. E. Barron-Casella E. A. Casella J. F.. (2012). Heme oxygenase-1 gene promoter polymorphism is associated with reduced incidence of acute chest syndrome among children with sickle cell disease. Blood 120, 3822–3828. 10.1182/blood-2011-06-361642 22966170 PMC3488892

[B22] Belcher J. D. Bryant C. J. Nguyen J. Bowlin P. R. Kielbik M. C. Bischof J. C.. (2003). Transgenic sickle mice have vascular inflammation. Blood 101, 3953–3959. 10.1182/blood-2002-10-3313 12543857

[B23] Belcher J. D. Chen C. Nguyen J. Milbauer L. Abdulla F. Alayash A. I.. (2014). Heme triggers TLR4 signaling leading to endothelial cell activation and vaso-occlusion in murine sickle cell disease. Blood 123, 377–390. 10.1182/blood-2013-04-495887 24277079 PMC3894494

[B24] Belcher J. D. Chen C. Nguyen J. Zhang P. Abdulla F. Nguyen P.. (2016). Control of oxidative stress and inflammation in sickle cell disease with the Nrf2 activator dimethyl fumarate. Antioxid. Redox Signal. [Epub ahead of print]. 10.1089/ars.2015.6571 26914345 PMC5421647

[B25] Belcher J. D. Mahaseth H. Welch T. E. Otterbein L. E. Hebbel R. P. Vercellotti G. M. (2006). Heme oxygenase-1 is a modulator of inflammation and vaso-occlusion in transgenic sickle mice. J. Clin. Invest. 116, 808–816. 10.1172/JCI26857 16485041 PMC1366501

[B26] Belcher J. D. Vineyard J. V. Bruzzone C. M. Chen C. Beckman J. D. Nguyen J.. (2010). Heme oxygenase-1 gene delivery by sleeping beauty inhibits vascular stasis in a murine model of sickle cell disease. J. Mol. Med. (Berl.) 88, 665–675. 10.1007/s00109-010-0613-6 20306336 PMC2877767

[B27] Bissell D. M. Hammaker L. Schmid R. (1972). Hemoglobin and erythrocyte catabolism in rat liver: the separate roles of parenchymal and sinusoidal cells. Blood 40, 812–822. 5083873

[B28] Bolisetty S. Zarjou A. Hull T. D. Traylor A. M. Perianayagam A. Joseph R.. (2015). Macrophage and epithelial cell H-ferritin expression regulates renal inflammation. Kidney Int. 88, 95–108. 10.1038/ki.2015.102 25874599 PMC4490000

[B29] Bone R. C. (1991). Let's agree on terminology: definitions of sepsis. Crit. Care Med. 19, 973–976. 10.1097/00003246-199107000-00024 1824030

[B30] Bonkowsky H. L. Tschudy D. P. Collins A. Doherty J. Bossenmaier I. Cardinal R.. (1971). Repression of the overproduction of porphyrin precursors in acute intermittent porphyria by intravenous infusions of hematin. Proc. Natl. Acad. Sci. U.S.A. 68, 2725–2729. 10.1073/pnas.68.11.2725 5288250 PMC389510

[B31] Bunn H. F. Jandl J. H. (1968). Exchange of heme among hemoglobins and between hemoglobin and albumin. J. Biol. Chem. 243, 465–475. 4966113

[B32] Burris T. P. (2008). Nuclear hormone receptors for heme: REV-ERBα and REV-ERBβ are ligand-regulated components of the mammalian clock. Mol. Endocrinol. 22, 1509–1520. 10.1210/me.2007-0519 18218725 PMC5419435

[B33] Calay D. Mason J. C. (2014). The multifunctional role and therapeutic potential of HO-1 in the vascular endothelium. Antioxid. Redox Signal. 20, 1789–1809. 10.1089/ars.2013.5659 24131232

[B34] Camus S. M. De Moraes J. A. Bonnin P. Abbyad P. Le Jeune S. Lionnet F.. (2015). Circulating cell membrane microparticles transfer heme to endothelial cells and trigger vasoocclusions in sickle cell disease. Blood 125, 3805–3814. 10.1182/blood-2014-07-589283 25827830 PMC4490297

[B35] Cao J. Peterson S. J. Sodhi K. Vanella L. Barbagallo I. Rodella L. F.. (2012). Heme oxygenase gene targeting to adipocytes attenuates adiposity and vascular dysfunction in mice fed a high-fat diet. Hypertension 60, 467–475. 10.1161/HYPERTENSIONAHA.112.193805 22753217 PMC3423899

[B36] Cao J. Sodhi K. Inoue K. Quilley J. Rezzani R. Rodella L.. (2011). Lentiviral-human heme oxygenase targeting endothelium improved vascular function in angiotensin II animal model of hypertension. Hum. Gene Ther. 22, 271–282. 10.1089/hum.2010.059 20836698 PMC3057195

[B37] Chakravarti R. Aulak K. S. Fox P. L. Stuehr D. J. (2010). GAPDH regulates cellular heme insertion into inducible nitric oxide synthase. Proc. Natl. Acad. Sci. U.S.A. 107, 18004–18009. 10.1073/pnas.1008133107 20921417 PMC2964200

[B38] Chance B. (1967). The reactivity of haemoproteins and cytochromes. Biochem. J. 103, 1–18. 10.1042/bj1030001 5340507 PMC1270361

[B39] Chauveau C. Bouchet D. Roussel J. C. Mathieu P. Braudeau C. Renaudin K.. (2002). Gene transfer of heme oxygenase-1 and carbon monoxide delivery inhibit chronic rejection. Am. J. Transplant. 2, 581–592. 10.1034/j.1600-6143.2002.20702.x 12201358

[B40] Chen G. Zhang D. Fuchs T. A. Manwani D. Wagner D. D. Frenette P. S. (2014). Heme-induced neutrophil extracellular traps contribute to the pathogenesis of sickle cell disease. Blood 123, 3818–3827. 10.1182/blood-2013-10-529982 24620350 PMC4055928

[B41] Cheung P. K. Stulp B. Immenschuh S. Borghuis T. Baller J. F. Bakker W. (1999). Is 100KF an isoform of hemopexin? Immunochemical characterization of the vasoactive plasma factor 100KF. J. Am. Soc. Nephrol. 10, 1700–1708. 10446937 10.1681/ASN.V1081700

[B42] Chiabrando D. Vinchi F. Fiorito V. Mercurio S. Tolosano E. (2014). Heme in pathophysiology: a matter of scavenging, metabolism and trafficking across cell membranes. Front. Pharmacol. 5:61. 10.3389/fphar.2014.00061 24782769 PMC3986552

[B43] Deuel J. W. Vallelian F. Schaer C. A. Puglia M. Buehler P. W. Schaer D. J. (2015). Different target specificities of haptoglobin and hemopexin define a sequential protection system against vascular hemoglobin toxicity. Free Radic. Biol. Med. 89, 931–943. 10.1016/j.freeradbiomed.2015.09.016 26475040

[B44] Devadas K. Dhawan S. (2006). Hemin activation ameliorates HIV-1 infection via heme oxygenase-1 induction. J. Immunol. 176, 4252–4257. 10.4049/jimmunol.176.7.4252 16547262

[B45] Doberer D. Haschemi A. Andreas M. Zapf T. C. Clive B. Jeitler M.. (2010). Haem arginate infusion stimulates haem oxygenase-1 expression in healthy subjects. Br. J. Pharmacol. 161, 1751–1762. 10.1111/j.1476-5381.2010.00990.x 20718734 PMC3010580

[B46] Dorresteijn M. J. Paine A. Zilian E. Fenten M. G. Frenzel E. Janciauskiene S.. (2015). Cell-type-specific downregulation of heme oxygenase-1 by lipopolysaccharide via Bach1 in primary human mononuclear cells. Free Radic. Biol. Med. 78, 224–232. 10.1016/j.freeradbiomed.2014.10.579 25463280

[B47] Dutra F. F. Alves L. S. Rodrigues D. Fernandez P. L. de Oliveira R. B. Golenbock D. T.. (2014). Hemolysis-induced lethality involves inflammasome activation by heme. Proc. Natl. Acad. Sci. U.S.A. 111, E4110–E4118. 10.1073/pnas.1405023111 25225402 PMC4191786

[B48] Dutra F. F. Bozza M. T. (2014). Heme on innate immunity and inflammation. Front. Pharmacol. 5:115. 10.3389/fphar.2014.00115 24904418 PMC4035012

[B49] Elphinstone R. E. Conroy A. L. Hawkes M. Hermann L. Namasopo S. Warren H. S.. (2016). Alterations in systemic extracellular heme and hemopexin are associated with adverse clinical outcomes in Ugandan children with severe malaria. J. Infect. Dis. 214, 1268–1275. 10.1093/infdis/jiw357 27515862 PMC5034960

[B50] Exner M. Minar E. Wagner O. Schillinger M. (2004). The role of heme oxygenase-1 promoter polymorphisms in human disease. Free Radic. Biol. Med. 37, 1097–1104. 10.1016/j.freeradbiomed.2004.07.008 15451051

[B51] Figueiredo R. T. Fernandez P. L. Mourao-Sa D. S. Porto B. N. Dutra F. F. Alves L. S.. (2007). Characterization of heme as activator of Toll-like receptor 4. J. Biol. Chem. 282, 20221–20229. 10.1074/jbc.M610737200 17502383

[B52] Frimat M. Tabarin F. Dimitrov J. D. Poitou C. Halbwachs-Mecarelli L. Fremeaux-Bacchi V. T.. (2013). Complement activation by heme as a secondary hit for atypical hemolytic uremic syndrome. Blood 122, 282–292. 10.1182/blood-2013-03-489245 23692858

[B53] Ghosh S. Adisa O. A. Chappa P. Tan F. Jackson K. A. Archer D. R. Ofori-Acquah. (2013). Extracellular hemin crisis triggers acute chest syndrome in sickle mice. J. Clin. Invest. 123, 4809–4820. 10.1172/JCI64578 24084741 PMC3809772

[B54] Girvan H. M. Munro A. W. (2013). Heme sensor proteins. J. Biol. Chem. 288, 13194–13203. 10.1074/jbc.R112.422642 23539616 PMC3650359

[B55] Glueck R. Green D. Cohen I. Ts'ao C. H. (1983). Hematin: unique effects of hemostasis. Blood 61, 243–249. 6821696

[B56] Goetsch C. A. Bissell D. M. (1986). Instability of hematin used in the treatment of acute hepatic porphyria. N. Engl. J. Med. 315, 235–238. 10.1056/NEJM198607243150406 3724815

[B57] Gotts J. E. Matthay M. A. (2016). Sepsis: pathophysiology and clinical management. BMJ 353:i1585. 10.1136/bmj.i1585 27217054

[B58] Gozzelino R. Jeney V. Soares M. P. (2010). Mechanisms of cell protection by heme oxygenase-1. Annu. Rev. Pharmacol. Toxicol. 50, 323–354. 10.1146/annurev.pharmtox.010909.105600 20055707

[B59] Gram M. Sveinsdottir S. Cinthio M. Sveinsdottir K. Hansson S. R. Morgelin M.. (2014). Extracellular hemoglobin - mediator of inflammation and cell death in the choroid plexus following preterm intraventricular hemorrhage. J. Neuroinflammation 11:200. 10.1186/s12974-014-0200-9 25441622 PMC4269927

[B60] Granick S. Sinclair P. Sassa S. Grieninger G. (1975). Effects by heme, insulin, and serum albumin on heme and protein synthesis in chick embryo liver cells cultured in a chemically defined medium, and a spectrofluorometric assay for porphyrin composition. J. Biol. Chem. 250, 9215–9225. 1238396

[B61] Graw J. A. Mayeur C. Rosales I. Liu Y. Sabbisetti V. S. Riley F. E.. (2016). Haptoglobin or hemopexin therapy prevents acute adverse effects of resuscitation after prolonged storage of red cells. Circulation 134, 945–960. 10.1161/CIRCULATIONAHA.115.019955 27515135 PMC5039096

[B62] Greil J. Verga-Falzacappa M. V. Echner N. E. Behnisch W. Bandapalli O. R. Pechanska P.. (2016). Mutating heme oxygenase-1 into a peroxidase causes a defect in bilirubin synthesis associated with microcytic anemia and severe hyperinflammation. Haematologica 101, e436–e439. 10.3324/haematol.2016.147090 27662012 PMC5394876

[B63] Griffiths E. Cortes A. Gilbert N. Stevenson P. MacDonald S. Pepper D. (1995). Haemoglobin-based blood substitutes and sepsis. Lancet 345, 158–160. 10.1016/S0140-6736(95)90168-X 7823671

[B64] Grosser N. Hemmerle A. Berndt G. Erdmann K. Hinkelmann U. Schurger S.. (2004). The antioxidant defense protein heme oxygenase 1 is a novel target for statins in endothelial cells. Free Radic. Biol. Med. 37, 2064–2071. 10.1016/j.freeradbiomed.2004.09.009 15544924

[B65] Hada H. Shiraki T. Watanabe-Matsui M. Igarashi K. (2014). Hemopexin-dependent heme uptake via endocytosis regulates the Bach1 transcription repressor and heme oxygenase gene activation. Biochim. Biophys. Acta 1840, 2351–2360. 10.1016/j.bbagen.2014.02.029 24613679

[B66] Haldar M. Kohyama M. So A. Y. Kc W. Wu X. Briseno C. G.. (2014). Heme-mediated SPI-C induction promotes monocyte differentiation into iron-recycling macrophages. Cell 156, 1223–1234. 10.1016/j.cell.2014.01.069 24630724 PMC4010949

[B67] Hamza I. Dailey H. A. (2012). One ring to rule them all: trafficking of heme and heme synthesis intermediates in the metazoans. Biochim. Biophys. Acta 1823, 1617–1632. 10.1016/j.bbamcr.2012.04.009 22575458 PMC3412874

[B68] Harvey J. W. Beutler E. (1982). Binding of heme by glutathione S-transferase: a possible role of the erythrocyte enzyme. Blood 60, 1227–1230. 7126873

[B69] Hebbel R. P. Morgan W. T. Eaton J. W. Hedlund B. E. (1988). Accelerated autoxidation and heme loss due to instability of sickle hemoglobin. Proc. Natl. Acad. Sci. U.S.A. 85, 237–241. 10.1073/pnas.85.1.237 3422420 PMC279519

[B70] Heinrich P. C. Castell J. V. Andus T. (1990). Interleukin-6 and the acute phase response. Biochem. J. 265, 621–636. 10.1042/bj2650621 1689567 PMC1133681

[B71] Horvath K. Varga C. Berko A. Posa A. Laszlo F. Whittle B. J. (2008). The involvement of heme oxygenase-1 activity in the therapeutic actions of 5-aminosalicylic acid in rat colitis. Eur. J. Pharmacol. 581, 315–323. 10.1016/j.ejphar.2007.12.004 18215658

[B72] Hvidberg V. Maniecki M. B. Jacobsen C. Hojrup P. Moller H. J. Moestrup S. K. (2005). Identification of the receptor scavenging hemopexin-heme complexes. Blood 106, 2572–2579. 10.1182/blood-2005-03-1185 15947085

[B73] Immenschuh S. Baumgart-Vogt E. Tan M. Iwahara S. Ramadori G. Fahimi H. D. (2003). Differential cellular and subcellular localization of heme-binding protein 23/peroxiredoxin I and heme oxygenase-1 in rat liver. J. Histochem. Cytochem. 51, 1621–1631. 10.1177/002215540305101206 14623930

[B74] Immenschuh S. Hinke V. Ohlmann A. Gifhorn-Katz S. Katz N. Jungermann K.. (1998a). Transcriptional activation of the haem oxygenase-1 gene by cGMP via a cAMP response element/activator protein-1 element in primary cultures of rat hepatocytes. Biochem. J. 334(Pt 1), 141–146. 10.1042/bj3340141 9693113 PMC1219672

[B75] Immenschuh S. Kietzmann T. Hinke V. Wiederhold M. Katz N. Muller-Eberhard U. (1998b). The rat heme oxygenase-1 gene is transcriptionally induced via the protein kinase A signaling pathway in rat hepatocyte cultures. Mol. Pharmacol. 53, 483–491. 9495815 10.1124/mol.53.3.483

[B76] Immenschuh S. Nagae Y. Satoh H. Baumann H. Muller-Eberhard U. (1994). The rat and human hemopexin genes contain an identical interleukin-6 response element that is not a target of CAAT enhancer-binding protein isoforms. J. Biol. Chem. 269, 12654–12661. 8175675

[B77] Immenschuh S. Ramadori G. (2000). Gene regulation of heme oxygenase-1 as a therapeutic target. Biochem. Pharmacol. 60, 1121–1128. 10.1016/S0006-2952(00)00443-3 11007950

[B78] Immenschuh S. Tan M. Ramadori G. (1999). Nitric oxide mediates the lipopolysaccharide dependent upregulation of the heme oxygenase-1 gene expression in cultured rat Kupffer cells. J. Hepatol. 30, 61–69. 10.1016/S0168-8278(99)80008-7 9927151

[B79] Ingram V. M. (1957). Gene mutations in human haemoglobin: the chemical difference between normal and sickle cell haemoglobin. Nature 180, 326–328. 10.1038/180326a0 13464827

[B80] Iwahara S. Satoh H. Song D. X. Webb J. Burlingame A. L. Nagae Y. (1995). Purification, characterization and cloning of a heme-binding protein (23kDa) in rat liver cytosol. Biochemistry 34, 13398–13406. 10.1021/bi00041a017 7577926

[B81] Janz D. R. Bastarache J. A. Sills G. Wickersham N. May A. K. Bernard G. R.. (2013). Association between haptoglobin, hemopexin and mortality in adults with sepsis. Crit. Care 17:R272. 10.1186/cc13108 24225252 PMC4056258

[B82] Jeney V. Balla G. Balla J. (2014). Red blood cell, hemoglobin and heme in the progression of atherosclerosis. Front. Physiol. 5:379. 10.3389/fphys.2014.00379 25324785 PMC4183119

[B83] Jeney V. Balla J. Yachie A. Varga Z. Vercellotti G. M. Eaton J. W.. (2002). Pro-oxidant and cytotoxic effects of circulating heme. Blood 100, 879–887. 10.1182/blood.V100.3.879 12130498

[B84] Kapturczak M. H. Wasserfall C. Brusko T. Campbell-Thompson M. Ellis T. M. Atkinson M. A.. (2004). Heme oxygenase-1 modulates early inflammatory responses: evidence from the heme oxygenase-1-deficient mouse. Am. J. Pathol. 165, 1045–1053. 10.1016/S0002-9440(10)63365-2 15331427 PMC1618611

[B85] Karnaukhova E. Krupnikova S. S. Rajabi M. Alayash A. I. (2012). Heme binding to human alpha-1 proteinase inhibitor. Biochim. Biophys. Acta 1820, 2020–2029. 10.1016/j.bbagen.2012.09.012 23000493

[B86] Keleku-Lukwete N. Suzuki M. Otsuki A. Tsuchida K. Katayama S. Hayashi M.. (2015). Amelioration of inflammation and tissue damage in sickle cell model mice by Nrf2 activation. Proc. Natl. Acad. Sci. U.S.A. 112, 12169–12174. 10.1073/pnas.1509158112 26371321 PMC4593117

[B87] Khechaduri A. Bayeva M. Chang H. C. Ardehali H. (2013). Heme levels are increased in human failing hearts. J. Am. Coll. Cardiol. 61, 1884–1893. 10.1016/j.jacc.2013.02.012 23500306 PMC3739715

[B88] Kordac V. Kozakova M. Martasek P. (1989). Changes of myocardial functions in acute hepatic porphyrias. Role of heme arginate administration. Ann. Med. 21, 273–276. 10.3109/07853898909149205 2551350

[B89] Kovtunovych G. Eckhaus M. A. Ghosh M. C. Ollivierre-Wilson H. Rouault T. A. (2010). Dysfunction of the heme recycling system in heme oxygenase 1-deficient mice: effects on macrophage viability and tissue iron distribution. Blood 116, 6054–6062. 10.1182/blood-2010-03-272138 20844238 PMC3031391

[B90] Kumar S. Bandyopadhyay U. (2005). Free heme toxicity and its detoxification systems in human. Toxicol. Lett. 157, 175–188. 10.1016/j.toxlet.2005.03.004 15917143

[B91] Kutty R. K. Maines M. D. (1981). Purification and characterization of biliverdin reductase from rat liver. J. Biol. Chem. 256, 3956–3962. 7217067

[B92] Larsen R. Gouveia Z. Soares M. P. Gozzelino R. (2012). Heme cytotoxicity and the pathogenesis of immune-mediated inflammatory diseases. Front. Pharmacol. 3:77. 10.3389/fphar.2012.00077 22586395 PMC3343703

[B93] Larsen R. Gozzelino R. Jeney V. Tokaji L. Bozza F. A. Japiassu A. M.. (2010). A central role for free heme in the pathogenesis of severe sepsis. Sci. Transl. Med. 2:51ra71. 10.1126/scitranslmed.3001118 20881280

[B94] Lee T. S. Chang C. C. Zhu Y. Shyy J. Y. (2004). Simvastatin induces heme oxygenase-1: a novel mechanism of vessel protection. Circulation 110, 1296–1302. 10.1161/01.CIR.0000140694.67251.9C 15337692

[B95] Lelubre C. Piagnerelli M. Vincent J. L. (2009). Association between duration of storage of transfused red blood cells and morbidity and mortality in adult patients: myth or reality? Transfusion 49, 1384–1394. 10.1111/j.1537-2995.2009.02211.x 19453985

[B96] Lever J. M. Boddu R. George J. F. Agarwal A. (2016). Heme oxygenase-1 in kidney health and disease. Antioxid. Redox Signal. 25, 165–183. 10.1089/ars.2016.6659 26906116 PMC4948210

[B97] Levere R. D. Gong Y. F. Kappas A. Bucher D. J. Wormser G. P. Abraham N. G. (1991). Heme inhibits human immunodeficiency virus 1 replication in cell cultures and enhances the antiviral effect of zidovudine. Proc. Natl. Acad. Sci. U.S.A. 88, 1756–1759. 10.1073/pnas.88.5.1756 2000384 PMC51103

[B98] Li C. Hossieny P. Wu B. J. Qawasmeh A. Beck K. Stocker R. (2007). Pharmacologic induction of heme oxygenase-1. Antioxid. Redox Signal. 9, 2227–2239. 10.1089/ars.2007.1783 17822367

[B99] Liem H. H. Noy N. Muller-Eberhard U. (1994). Studies on the efflux of heme from biological membranes. Biochim. Biophys. Acta 1194, 264–270. 10.1016/0005-2736(94)90308-5 7918539

[B100] Lin T. Kwak Y. H. Sammy F. He P. Thundivalappil S. Sun G.. (2010). Synergistic inflammation is induced by blood degradation products with microbial Toll-like receptor agonists and is blocked by hemopexin. J. Infect. Dis. 202, 624–632. 10.1086/654929 20617898 PMC2932749

[B101] Lin T. Liu J. Huang F. Van Engelen T. S. Thundivalappil S. R. Riley F. E. (2016). Purified and recombinant hemopexin: protease activity and effect on neutrophil chemotaxis. Mol. Med. 22, 22–31. 10.2119/molmed.2016.00006 PMC5004720 26772775

[B102] Lin T. Maita D. Thundivalappil S. R. Riley F. E. Hambsch J. Van Marter L. J.. (2015). Hemopexin in severe inflammation and infection: mouse models and human diseases. Crit. Care 19:166. 10.1186/s13054-015-0885-x 25888135 PMC4424824

[B103] Little H. N. Neilands J. B. (1960). Binding of haematin by human serum albumin. Nature 188, 913–915. 10.1038/188913a0 13762740

[B104] Lundvig D. M. Immenschuh S. Wagener F. A. (2012). Heme oxygenase, inflammation, and fibrosis: the good, the bad, and the ugly? Front. Pharmacol. 3:81. 10.3389/fphar.2012.00081 22586396 PMC3345581

[B105] Ma B. Day J. P. Phillips H. Slootsky B. Tolosano E. Dore S. (2016). Deletion of the hemopexin or heme oxygenase-2 gene aggravates brain injury following stroma-free hemoglobin-induced intracerebral hemorrhage. J. Neuroinflammation 13:26. 10.1186/s12974-016-0490-1 26831741 PMC4736638

[B106] Maines M. D. (1997). The heme oxygenase system: a regulator of second messenger gases. Annu. Rev. Pharmacol. Toxicol. 37, 517–554. 10.1146/annurev.pharmtox.37.1.517 9131263

[B107] Maines M. D. Kappas A. (1974). Cobalt induction of hepatic heme oxygenase; with evidence that cytochrome P-450 is not essential for this enzyme activity. Proc. Natl. Acad. Sci. U.S.A. 71, 4293–4297. 10.1073/pnas.71.11.4293 4530983 PMC433868

[B108] Martin D. Rojo A. I. Salinas M. Diaz R. Gallardo G. Alam J.. (2004). Regulation of heme oxygenase-1 expression through the phosphatidylinositol 3-kinase/Akt pathway and the Nrf2 transcription factor in response to the antioxidant phytochemical carnosol. J. Biol. Chem. 279, 8919–8929. 10.1074/jbc.M309660200 14688281

[B109] Mendonca V. R. Luz N. F. Santos N. J. Borges V. M. Goncalves M. S. Andrade B. B.. (2012). Association between the haptoglobin and heme oxygenase 1 genetic profiles and soluble CD163 in susceptibility to and severity of human malaria. Infect. Immun. 80, 1445–1454. 10.1128/IAI.05933-11 22290142 PMC3318432

[B110] Mense S. M. Zhang L. (2006). Heme: a versatile signaling molecule controlling the activities of diverse regulators ranging from transcription factors to MAP kinases. Cell Res. 16, 681–692. 10.1038/sj.cr.7310086 16894358

[B111] Mestas J. Hughes C. C. (2004). Of mice and not men: differences between mouse and human immunology. J. Immunol. 172, 2731–2738. 10.4049/jimmunol.172.5.2731 14978070

[B112] Miller L. H. Baruch D. I. Marsh K. Doumbo O. K. (2002). The pathogenic basis of malaria. Nature 415, 673–679. 10.1038/415673a 11832955

[B113] Miyazaki T. Kirino Y. Takeno M. Samukawa S. Hama M. Tanaka M.. (2010). Expression of heme oxygenase-1 in human leukemic cells and its regulation by transcriptional repressor Bach1. Cancer Sci. 101, 1409–1416. 10.1111/j.1349-7006.2010.01550.x 20345481 PMC11159765

[B114] Mosser D. M. Edwards J. P. (2008). Exploring the full spectrum of macrophage activation. Nat. Rev. Immunol. 8, 958–969. 10.1038/nri2448 19029990 PMC2724991

[B115] Motterlini R. Foresti R. (2014). Heme oxygenase-1 as a target for drug discovery. Antioxid. Redox Signal. 20, 1810–1826. 10.1089/ars.2013.5658 24180608

[B116] Muller Eberhard U. (1970). Hemopexin. N. Engl. J. Med. 283, 1090–1094. 10.1056/NEJM197011122832007 4921465

[B117] Muller-Eberhard U. Cleve H. (1963). Immunoelectrophoretic studies of the beta1-haem-binding globulin (haemopexin) in hereditary haemolytic disorders. Nature 197, 602–603. 10.1038/197602a0 13936558

[B118] Muller-Eberhard U. Javid J. Liem H. H. Hanstein A. Hanna M. (1968). Plasma concentrations of hemopexin, haptoglobin and heme in patients with various hemolytic diseases. Blood 32, 811–815. 5687939

[B119] Muller Eberhard U. Nikkilä H. (1989). Transport of tetrapyrroles by proteins. Semin. Hematol. 26, 86–104. 2658093

[B120] Mustajoki P. Tenhunen R. Tokola O. Gothoni G. (1986). Haem arginate in the treatment of acute hepatic porphyrias. Br. Med. J. (Clin. Res. Ed.) 293, 538–539. 10.1136/bmj.293.6546.538-a 3092906 PMC1341312

[B121] Nagy E. Eaton J. W. Jeney V. Soares M. P. Varga Z. Galajda Z.. (2010). Red cells, hemoglobin, heme, iron, and atherogenesis. Arterioscler. Thromb. Vasc. Biol. 30, 1347–1353. 10.1161/ATVBAHA.110.206433 20378845 PMC2893144

[B122] Nakamichi I. Habtezion A. Zhong B. Contag C. H. Butcher E. C. Omary M. B. (2005). Hemin-activated macrophages home to the pancreas and protect from acute pancreatitis via heme oxygenase-1 induction. J. Clin. Invest. 115, 3007–3014. 10.1172/JCI24912 16239966 PMC1257535

[B123] Nath K. A. Balla G. Vercellotti G. M. Balla J. Jacob H. S. Levitt M. D.. (1992). Induction of heme oxygenase is a rapid, protective response in rhabdomyolysis in the rat. J. Clin. Invest. 90, 267–270. 10.1172/JCI115847 1634613 PMC443091

[B124] Nath K. A. Balla J. Croatt A. J. Vercellotti G. M. (1995). Heme protein-mediated renal injury: a protective role for 21-aminosteroids *in vitro* and *in vivo*. Kidney Int. 47, 592–602. 10.1038/ki.1995.75 7723246

[B125] Nath K. A. Katusic Z. S. (2012). Vasculature and kidney complications in sickle cell disease. J. Am. Soc. Nephrol. 23, 781–784. 10.1681/ASN.2011101019 22440903 PMC3338300

[B126] Ndisang J. F. Lane N. Jadhav A. (2009). The heme oxygenase system abates hyperglycemia in Zucker diabetic fatty rats by potentiating insulin-sensitizing pathways. Endocrinology 150, 2098–2108. 10.1210/en.2008-0239 19106228

[B127] Otterbein L. Sylvester S. L. Choi A. M. (1995). Hemoglobin provides protection against lethal endotoxemia in rats: the role of heme oxygenase-1. Am. J. Respir. Cell Mol. Biol. 13, 595–601. 10.1165/ajrcmb.13.5.7576696 7576696

[B128] Paine A. Eiz-Vesper B. Blasczyk R. Immenschuh S. (2010). Signaling to heme oxygenase-1 and its anti-inflammatory therapeutic potential. Biochem. Pharmacol. 80, 1895–1903. 10.1016/j.bcp.2010.07.014 20643109

[B129] Pamplona A. Ferreira A. Balla J. Jeney V. Balla G. Epiphanio S.. (2007). Heme oxygenase-1 and carbon monoxide suppress the pathogenesis of experimental cerebral malaria. Nat. Med. 13, 703–710. 10.1038/nm1586 17496899

[B130] Paoli M. Anderson B. F. Baker H. M. Morgan W. T. Smith A. Baker E.. (1999). Crystal structure of hemopexin reveals a novel high-affinity heme site formed between two β-propeller domains. Nat. Struct. Biol. 6, 926–931. 10.1038/13294 10504726

[B131] Paul G. Bataille F. Obermeier F. Bock J. Klebl F. Strauch U.. (2005). Analysis of intestinal haem-oxygenase-1 (HO-1) in clinical and experimental colitis. Clin. Exp. Immunol. 140, 547–555. 10.1111/j.1365-2249.2005.02775.x 15932518 PMC1809385

[B132] Pechlaner R. Willeit P. Summerer M. Santer P. Egger G. Kronenberg F.. (2015). Heme oxygenase-1 gene promoter microsatellite polymorphism is associated with progressive atherosclerosis and incident cardiovascular disease. Arterioscler. Thromb. Vasc. Biol. 35, 229–236. 10.1161/ATVBAHA.114.304729 25359861 PMC4317265

[B133] Petersen B. Ramackers W. Lucas-Hahn A. Lemme E. Hassel P. Queisser A. L.. (2011). Transgenic expression of human heme oxygenase-1 in pigs confers resistance against xenograft rejection during *ex vivo* perfusion of porcine kidneys. Xenotransplantation 18, 355–368. 10.1111/j.1399-3089.2011.00674.x 22168142

[B134] Peterson S. J. Kim D. H. Li M. Positano V. Vanella L. Rodella L. F.. (2009). The L-4F mimetic peptide prevents insulin resistance through increased levels of HO-1, pAMPK, and pAKT in obese mice. J. Lipid Res. 50, 1293–1304. 10.1194/jlr.M800610-JLR200 19224872 PMC2694329

[B135] Pober J. S. Min W. Bradley J. R. (2009). Mechanisms of endothelial dysfunction, injury, and death. Annu. Rev. Pathol. 4, 71–95. 10.1146/annurev.pathol.4.110807.092155 18754744

[B136] Poli V. Mancini F. P. Cortese R. (1990). IL-6DBP, a nuclear protein involved in interleukin-6 signal transduction, defines a new family of leucine zipper proteins related to C/EBP. Cell 63, 643–653. 10.1016/0092-8674(90)90459-R 2171780

[B137] Ponka P. (1997). Tissue-specific regulation of iron metabolism and heme synthesis: distinct control mechanisms in erythroid cells. Blood 89, 1–25. 8978272

[B138] Ponka P. (1999). Cell biology of heme. Am. J. Med. Sci. 318, 241–256. 10.1016/S0002-9629(15)40628-7 10522552

[B139] Poss K. D. Thomas M. J. Ebralidze A. K. O'Dell T. J. Tonegawa S. (1995). Hippocampal long-term potentiation is normal in heme oxygenase-2 mutant mice. Neuron 15, 867–873. 10.1016/0896-6273(95)90177-9 7576635

[B140] Poss K. D. Tonegawa S. (1997a). Reduced stress defense in heme oxygenase 1-deficient cells. Proc. Natl. Acad. Sci. U.S.A. 94, 10925–10930. 10.1073/pnas.94.20.10925 9380736 PMC23533

[B141] Poss K. D. Tonegawa S. (1997b). Heme oxygenase 1 is required for mammalian iron reutilization. Proc. Natl. Acad. Sci. U.S.A. 94, 10919–10924. 10.1073/pnas.94.20.10919 9380735 PMC23531

[B142] Potter D. Chroneos Z. C. Baynes J. W. Sinclair P. R. Gorman N. Liem H. H.. (1993). *In vivo* fate of hemopexin and heme-hemopexin complexes in the rat. Arch. Biochem. Biophys. 300, 98–104. 10.1006/abbi.1993.1014 8424694

[B143] Reeder B. J. (2010). The redox activity of hemoglobins: from physiologic functions to pathologic mechanisms. Antioxid. Redox Signal. 13, 1087–1123. 10.1089/ars.2009.2974 20170402

[B144] Rifkind J. M. Nagababu E. (2013). Hemoglobin redox reactions and red blood cell aging. Antioxid. Redox Signal. 18, 2274–2283. 10.1089/ars.2012.4867 23025272 PMC3638511

[B145] Roumenina L. T. Rayes J. Lacroix-Desmazes S. Dimitrov J. D. (2016). Heme: modulator of plasma systems in hemolytic diseases. Trends Mol. Med. 22, 200–213. 10.1016/j.molmed.2016.01.004 26875449

[B146] Ryter S. W. Alam J. Choi A. M. (2006). Heme oxygenase-1/carbon monoxide: from basic science to therapeutic applications. Physiol. Rev. 86, 583–650. 10.1152/physrev.00011.2005 16601269

[B147] Ryter S. W. Tyrrell R. M. (2000). The heme synthesis and degradation pathways: role in oxidant sensitivity. Heme oxygenase has both pro- and antioxidant properties. Free Radic. Biol. Med. 28, 289–309. 10.1016/S0891-5849(99)00223-3 11281297

[B148] Sadrzadeh S. M. Anderson D. K. Panter S. S. Hallaway P. E. Eaton J. W. (1987). Hemoglobin potentiates central nervous system damage. J. Clin. Invest. 79, 662–664. 10.1172/JCI112865 3027133 PMC424162

[B149] Saragih H. Zilian E. Jaimes Y. Paine A. Figueiredo C. Eiz-Vesper B.. (2014). PECAM-1-dependent heme oxygenase-1 regulation via an Nrf2-mediated pathway in endothelial cells. Thromb. Haemost. 111, 1077–1088. 10.1160/TH13-11-0923 24500083

[B150] Satoh T. Satoh H. Iwahara S. Hrkal Z. Peyton D. H. Muller-Eberhard U. (1994). Roles of heme iron-coordinating histidine residues of human hemopexin expressed in baculovirus-infected insect cells. Proc. Natl. Acad. Sci. U.S.A. 91, 8423–8427. 10.1073/pnas.91.18.8423 8078898 PMC44618

[B151] Sawicki K. T. Shang M. Wu R. Chang H. C. Khechaduri A. Sato T.. (2015). Increased Heme Levels in the Heart Lead to Exacerbated Ischemic Injury. J. Am. Heart Assoc. 4:e002272. 10.1161/JAHA.115.002272 26231844 PMC4599478

[B152] Schaedler R. W. Dubos R. J. (1961). The susceptibility of mice to bacterial endotoxins. J. Exp. Med. 113, 559–570. 10.1084/jem.113.3.559 13747161 PMC2137366

[B153] Schaer D. J. Buehler P. W. Alayash A. I. Belcher J. D. Vercellotti G. M. (2012). Hemolysis and free hemoglobin revisited: exploring hemoglobin and hemin scavengers as a novel class of therapeutic proteins. Blood 121, 1276–1284. 10.1182/blood-2012-11-451229 23264591 PMC3578950

[B154] Schaer D. J. Vinchi F. Ingoglia G. Tolosano E. Buehler P. W. (2014). Haptoglobin, hemopexin, and related defense pathways-basic science, clinical perspectives, and drug development. Front. Physiol. 5:415. 10.3389/fphys.2014.00415 25389409 PMC4211382

[B155] Seixas E. Gozzelino R. Chora A. Ferreira A. Silva G. Larsen R.. (2009). Heme oxygenase-1 affords protection against noncerebral forms of severe malaria. Proc. Natl. Acad. Sci. U.S.A. 106, 15837–15842. 10.1073/pnas.0903419106 19706490 PMC2728109

[B156] Seok J. Warren H. S. Cuenca A. G. Mindrinos M. N. Baker H. V. Xu W.. (2013). Genomic responses in mouse models poorly mimic human inflammatory diseases. Proc. Natl. Acad. Sci. U.S.A. 110, 3507–3512. 10.1073/pnas.1222878110 23401516 PMC3587220

[B157] Shen Y. Ward N. C. Hodgson J. M. Puddey I. B. Wang Y. Zhang D.. (2013). Dietary quercetin attenuates oxidant-induced endothelial dysfunction and atherosclerosis in apolipoprotein E knockout mice fed a high-fat diet: a critical role for heme oxygenase-1. Free Radic. Biol. Med. 65, 908–915. 10.1016/j.freeradbiomed.2013.08.185 24017971

[B158] Sikorski E. M. Hock T. Hill-Kapturczak N. Agarwal A. (2004). The story so far: molecular regulation of the heme oxygenase-1 gene in renal injury. Am. J. Physiol. Renal Physiol. 286, F425–F441. 10.1152/ajprenal.00297.2003 14761930

[B159] Simionatto C. S. Cabal R. Jones R. L. Galbraith R. A. (1988). Thrombophlebitis and disturbed hemostasis following administration of intravenous hematin in normal volunteers. Am. J. Med. 85, 538–540. 10.1016/S0002-9343(88)80092-5 3177402

[B160] Smith A. McCulloh R. J. (2015). Hemopexin and haptoglobin: allies against heme toxicity from hemoglobin not contenders. Front. Physiol. 6:187 10.3389/fphys.2015.00187 26175690 PMC4485156

[B161] Soares M. P. Bozza M. T. (2016). Red alert: labile heme is an alarmin. Curr. Opin. Immunol. 38, 94–100. 10.1016/j.coi.2015.11.006 26741528

[B162] Solar I. Muller-Eberhard U. Shviro Y. Shaklai N. (1991). Long-term intercalation of residual hemin in erythrocyte membranes distorts the cell. Biochim. Biophys. Acta 1062, 51–58. 10.1016/0005-2736(91)90334-5 1998709

[B163] Son Y. Lee J. H. Chung H. T. Pae H. O. (2013). Therapeutic roles of heme oxygenase-1 in metabolic diseases: curcumin and resveratrol analogues as possible inducers of heme oxygenase-1. Oxid. Med. Cell Longev. 2013:639541. 10.1155/2013/639541 24101950 PMC3786516

[B164] Spiller F. Costa C. Souto F. O. Vinchi F. Mestriner F. L. Laure H. J.. (2011). Inhibition of neutrophil migration by hemopexin leads to increased mortality due to sepsis in mice. Am. J. Respir. Crit. Care Med. 183, 922–931. 10.1164/rccm.201002-0223OC 20971829

[B165] Stapley R. Rodriguez C. Oh J. Y. Honavar J. Brandon A. Wagener B. M.. (2015). Red blood cell washing, nitrite therapy, and antiheme therapies prevent stored red blood cell toxicity after trauma-hemorrhage. Free Radic. Biol. Med. 85, 207–218. 10.1016/j.freeradbiomed.2015.04.025 25933588 PMC4508223

[B166] Taketani S. Adachi Y. Kohno H. Ikehara S. Tokunaga R. Ishii T. (1998). Molecular characterization of a newly identified heme-binding protein induced during differentiation of murine erythroleukemia cells. J. Biol. Chem. 273, 31388–31394. 10.1074/jbc.273.47.31388 9813049

[B167] Tenhunen R. Marver H. S. Schmid R. (1968). The enzymatic conversion of heme to bilirubin by microsomal heme oxygenase. Proc. Natl. Acad. Sci. U.S.A. 61, 748–755. 10.1073/pnas.61.2.748 4386763 PMC225223

[B168] Thomas R. A. Czopek A. Bellamy C. O. McNally S. J. Kluth D. C. Marson L. P. (2016). Hemin preconditioning upregulates heme oxygenase-1 in deceased donor renal transplant recipients: a randomized, controlled, phase IIB trial. Transplantation 100, 176–183. 10.1097/TP.0000000000000770 26680374

[B169] Tolosano E. Altruda F. (2002). Hemopexin: structure, function, and regulation. DNA Cell Biol. 21, 297–306. 10.1089/104454902753759717 12042069

[B170] Tolosano E. Fagoonee S. Hirsch E. Berger F. G. Baumann H. Silengo L.. (2002). Enhanced splenomegaly and severe liver inflammation in haptoglobin/hemopexin double-null mice after acute hemolysis. Blood 100, 4201–4208. 10.1182/blood-2002-04-1270 12393471

[B171] Tolosano E. Fagoonee S. Morello N. Vinchi F. Fiorito V. (2010). Heme scavenging and the other facets of hemopexin. Antioxid. Redox Signal. 12, 305–320. 10.1089/ars.2009.2787 19650691

[B172] Tolosano E. Hirsch E. Patrucco E. Camaschella C. Navone R. Silengo L.. (1999). Defective recovery and severe renal damage after acute hemolysis in hemopexin-deficient mice. Blood 94, 3906–3914. 10572107

[B173] Tracz M. J. Alam J. Nath K. A. (2007). Physiology and pathophysiology of heme: implications for kidney disease. J. Am. Soc. Nephrol. 18, 414–420. 10.1681/ASN.2006080894 17229906

[B174] Trakshel G. M. Kutty R. K. Maines M. D. (1986). Purification and characterization of the major constitutive form of testicular heme oxygenase. The noninducible isoform. J. Biol. Chem. 261, 11131–11137. 3525562

[B175] True A. L. Olive M. Boehm M. San H. Westrick R. J. Raghavachari N.. (2007). Heme oxygenase-1 deficiency accelerates formation of arterial thrombosis through oxidative damage to the endothelium, which is rescued by inhaled carbon monoxide. Circ. Res. 101, 893–901. 10.1161/CIRCRESAHA.107.158998 17885218

[B176] Tzima S. Victoratos P. Kranidioti K. Alexiou M. Kollias G. (2009). Myeloid heme oxygenase-1 regulates innate immunity and autoimmunity by modulating IFN-β production. J. Exp. Med. 206, 1167–1179. 10.1084/jem.20081582 19398754 PMC2715044

[B177] Vercellotti G. M. Zhang P. Nguyen J. Abdulla F. Chen C. Nguyen P.. (2016). Hepatic overexpression of hemopexin inhibits inflammation and vascular stasis in murine models of sickle cell disease. Mol. Med. 22, 437–451. 10.2119/molmed.2016.00063 27451971 PMC5082291

[B178] Vincent S. H. (1989). Oxidative effects of heme and porphyrins on proteins and lipids. Semin. Hematol. 26, 105–113. 2658086

[B179] Vincent S. H. Grady R. W. Shaklai N. Snider J. M. Muller-Eberhard U. (1988). The influence of heme-binding proteins in heme-catalyzed oxidations. Arch. Biochem. Biophys. 265, 539–550. 10.1016/0003-9861(88)90159-2 3421724

[B180] Vincent S. H. Muller Eberhard U. (1985). A protein of the Z class of liver cytosolic proteins in the rat that preferentially binds heme. J. Biol. Chem. 260, 14521–14528. 4055786

[B181] Vinchi F. De Franceschi L. Ghigo A. Townes T. Cimino J. Silengo L.. (2013). Hemopexin therapy improves cardiovascular function by preventing heme-induced endothelial toxicity in mouse models of hemolytic diseases. Circulation 127, 1317–1329. 10.1161/CIRCULATIONAHA.112.130179 23446829

[B182] Vinchi F. Gastaldi S. Silengo L. Altruda F. Tolosano E. (2008). Hemopexin prevents endothelial damage and liver congestion in a mouse model of heme overload. Am. J. Pathol. 173, 289–299. 10.2353/ajpath.2008.071130 18556779 PMC2438305

[B183] Vinchi F. Costa da Silva M. Ingoglia G. Petrillo S. Brinkman N. Zuercher A.. (2016). Hemopexin therapy reverts heme-induced proinflammatory phenotypic switching of macrophages in a mouse model of sickle cell disease. Blood 127, 473–486. 10.1182/blood-2015-08-663245 26675351 PMC4850229

[B184] Wagener E. Feldman T. de Witte T. Abraham N. G. (1997). Heme induces the expression of adhesion molecules ICAM-1, VCAM-1, and E selectin in vascular endothelial cells. Proc. Soc. Exp. Biol. Med. 216, 456–463. 10.3181/00379727-216-44197 9402154

[B185] Wagener F. A. Eggert A. Boerman O. C. Oyen W. J. Verhofstad A. Abraham N. G.. (2001). Heme is a potent inducer of inflammation in mice and is counteracted by heme oxygenase. Blood 98, 1802–1811. 10.1182/blood.V98.6.1802 11535514

[B186] Wagener F. A. Volk H. D. Willis D. Abraham N. G. Soares M. P. Adema G. J.. (2003). Different faces of the heme-heme oxygenase system in inflammation. Pharmacol. Rev. 55, 551–571. 10.1124/pr.55.3.5 12869663

[B187] Wang D. Cortes-Puch I. Sun J. Solomon S. B. Kanias T. Remy K. E.. (2012a). Transfusion of older stored blood worsens outcomes in canines depending on the presence and severity of pneumonia. Transfusion 54, 1712–1724. 10.1111/trf.12607 24588210 PMC4214924

[B188] Wang D. Sun J. Solomon S. B. Klein H. G. Natanson C. (2012b). Transfusion of older stored blood and risk of death: a meta-analysis. Transfusion 52, 1184–1195. 10.1111/j.1537-2995.2011.03466.x 22188419 PMC3883449

[B189] Warren H. S. Tompkins R. G. Moldawer L. L. Seok J. Xu W. Mindrinos M. N. (2015). Mice are not men. Proc. Natl. Acad. Sci. U.S.A. 112:E345 10.1073/pnas.1414857111 25540422 PMC4313842

[B190] Wei Q. Hill W. D. Su Y. Huang S. Dong Z. (2011). Heme oxygenase-1 induction contributes to renoprotection by G-CSF during rhabdomyolysis-associated acute kidney injury. Am. J. Physiol. Renal Physiol. 301, F162–F170. 10.1152/ajprenal.00438.2010 21511696 PMC3129892

[B191] Wijayanti N. Katz N. Immenschuh S. (2004). Biology of heme in health and disease. Curr. Med. Chem. 11, 981–986. 10.2174/0929867043455521 15078160

[B192] Wijayanti N. Kietzmann T. Immenschuh S. (2005). Heme oxygenase-1 gene activation by the NAD(P)H oxidase inhibitor 4-(2-aminoethyl) benzenesulfonyl fluoride via a protein kinase B, p38-dependent signaling pathway in monocytes. J. Biol. Chem. 280, 21820–21829. 10.1074/jbc.M502943200 15833736

[B193] Yachie A. Niida Y. Wada T. Igarashi N. Kaneda H. Toma T.. (1999). Oxidative stress causes enhanced endothelial cell injury in human heme oxygenase-1 deficiency. J. Clin. Invest. 103, 129–135. 10.1172/JCI4165 9884342 PMC407858

[B194] Yamada N. Yamaya M. Okinaga S. Nakayama K. Sekizawa K. Shibahara S.. (2000). Microsatellite polymorphism in the heme oxygenase-1 gene promoter is associated with susceptibility to emphysema. Am. J. Hum. Genet. 66, 187–195. 10.1086/302729 10631150 PMC1288325

[B195] Yuan X. Rietzschel N. Kwon H. Walter Nuno A. B. Hanna D. A. Phillips J. D.. (2016). Regulation of intracellular heme trafficking revealed by subcellular reporters. Proc. Natl. Acad. Sci. U.S.A. 113, E5144–E5152. 10.1073/pnas.1609865113 27528661 PMC5024633

[B196] Zarjou A. Bolisetty S. Joseph R. Traylor A. Apostolov E. O. Arosio P.. (2013). Proximal tubule H-ferritin mediates iron trafficking in acute kidney injury. J. Clin. Invest. 123, 4423–4434. 10.1172/JCI67867 24018561 PMC3784534

[B197] Zhong H. Bao W. Friedman D. Yazdanbakhsh K. (2014). Hemin controls T cell polarization in sickle cell alloimmunization. J. Immunol. 193, 102–110. 10.4049/jimmunol.1400105 24879794 PMC4068268

